# Application of UHPLC-MS/MS method to monitor the occurrence of sulfonamides and their transformation products in soil in Silesia, Poland

**DOI:** 10.1007/s11356-023-30146-y

**Published:** 2023-10-16

**Authors:** Klaudia Stando, Joanna Wilk, Agata Jakóbik-Kolon, Ewa Felis, Sylwia Bajkacz

**Affiliations:** 1 Faculty of Chemistry, Department of Inorganic Chemistry, Analytical Chemistry and Electrochemistry, Silesian University of Technology, B. Krzywoustego 6 Str, 44-100 Gliwice, Poland; 2 Biotechnology Centre, Silesian University of Technology, B. Krzywoustego 8 Str, 44-100 Gliwice, Poland; 3 Faculty of Power and Environmental Engineering, Environmental Biotechnology Department, Silesian University of Technology, Akademicka 2 Str, 44-100 Gliwice, Poland

**Keywords:** Sulfonamides, Transformation products, Organic carbon, Environmental monitoring, Soil pollution, Urban areas, LC-MS/MS-sulfonamides recovery from soil

## Abstract

Sulfonamides circulating in the environment lead to disturbances in food chains and local ecosystems, but most importantly contribute to development of resistance genes, which generate problems with multidrug-resistant bacterial infections treatment. In urban areas, sources of sulfonamide distribution in soils have received comparatively less attention in contrast to rural regions, where animal-derived manure, used as a natural fertilizer, is considered the main source. The aim of this study was to determine eight sulfonamides (sulfadiazine, sulfamerazine, sulfamethazine, sulfamethizole, sulfamethoxazole, sulfapyridine, sulfathiazole, and sulfisoxazole) in environmental soil samples collected from urbanized regions in Silesian Voivodeship with increased animal activity. These soils were grouped according to the organic carbon content. It was necessary to develop versatile and efficient extraction and determination method to analyze selected sulfonamides in various soil types. The developed LC–MS/MS method for sulfonamides analyzing was validated. The obtained recoveries exceeded 45% for soil with medium organic carbon content and 88% for sample with a very low organic carbon content (arenaceous quartz). The obtained results show the high impact of organic matter on analytes adsorption in soil, which influences recovery. All eight sulfa drugs were determined in environmental samples in the concentration range 1.5–10.5 ng g^-1^. The transformation products of the analytes were also identified, and 29 transformation products were detected in 24 out of 27 extracts from soil samples.

**Supplementary Information:**

The online version contains supplementary material available at 10.1007/s11356-023-30146-y.

## Introduction

Sulfonamides (SAs) are a group of compounds with a broad spectrum of activity against bacteria and protozoa of the genera *Toxoplasma* and *Plasmodium*. SAs are analogs of para-aminobenzoic acid (SA core), the activity of which depends on the type of amine attached to the SA core (Yousef et al. 2018). The widespread and long-term use of SAs in medicine has greatly affected the environment by contaminating surface waters, groundwater, and soil (Sacher et al. 2001; Martínez-Carballo et al. 2007). The mobility and availability of SAs for plants in the water-soil system are controlled by the sorption of components onto the soil and their stability (Kodešová et al. 2015). The adsorption of SAs on soil particles increases with the aromaticity and electronegativity of the substituent attached to the SA core (Thiele-Bruhn et al. 2004). In a study carried out on 13 types of soil, it was found that the sorption of SAs strongly depends on soil properties, such as the content of organic carbon (OC) (Leal et al. 2013). Soil pH may also influence on the sorption of selected SAs. For example, the sulfamethazine (SFM) sorption coefficient depends on the soil pH, but it is not the dominant factor (Lertpaitoonpan et al. 2009).

Primary sources of SAs in soil environment are manure or slurry (Pan et al. 2014; Gu et al. 2019). Assessment of the fate and persistence of various SAs after releasing into soil has been the subject of research over the last 20 years (Díaz-Cruz et al. 2003; Yang et al. 2009; Wu et al. 2012; Albero et al. 2018). The type of soil, the type of fertilizers used, and the presence of plants significantly influence the dispersion and accumulation of SAs in the soil environment (Leal et al. 2013; Hou et al. 2015). It was found that adding manure to soil may slightly affect the rate of dispersion of SMX, and that a soil’s OC content and biological activity significantly affect the stability of SMX in soil (Wu et al. 2012). SAs are easily leached from soils with low OC content and high pH (Wang et al. 2015; Pan and Chu 2017). The occurrence of SAs in soils might also be influenced by wastewater discharges, although it has not been definitively proven (Yi et al. 2019a). Nevertheless, topic remains scarcely discussed in literature (García-Galán et al. 2013). Once introduced into the soil, SAs are transformed into transformation products (TPs). SAs are mainly biodegradable due to the action of heterotrophic microorganisms, e.g., denitrifiers and, to a lesser extent, due to abiotic degradation in the soil (Bílková et al. 2019). Within the literature, the most described degradation reactions of SAs in the environment are acetylation, hydroxylation, nitration, nitrosation, glucosidation and glucuronidation. However, it is possible for multiple transformation reactions to occur simultaneously, including those originating from human metabolism (Majewsky et al. 2015). The most frequently documented TPs of SAs are N4-acetylated forms, which can convert back to parent form (Li et al. 2021). The pathways of SAs transformation under environmental conditions are not fully understood, and most of the papers dealing with the topic focus on the degradation of SMX (Barbieri et al. 2012; Koba et al. 2017; Wang and Wang 2018) and SFD (Yang et al. 2009). The residues of SAs and their TPs are potentially harmful to humans. Based on the toxicity tests of SAs, it was found that their residues in aquatic environment showed an additive toxic effect for the tiny vascular plants, as e.g., *Lemna minor* (Białk-Bielińska et al. 2017). Additionally, SAs presence in the environment not only contributes to antibiotic-resistant pathogens development, but also to the spread of resistance genes (Ayukekbong et al. 2017; Tao et al. 2022).

Hoff et al. noted the difficulties in extracting SAs from biosolid samples (soil, manure, or sediment) (Hoff et al. 2016). Due to the complexity of the soil matrix and its variable composition, it is necessary to use multi-stage procedures to extract and clean samples before analysis (Hoff et al. 2016). The most common methods for extracting SAs from solid samples are solid-liquid extraction (SLE) in combination with solid-phase extraction (SPE) (Hu et al. 2012; Moyo and Tavengwa 2022). Despite the many developed and validated soil preparation procedures, they are characterized by low reproducibility. Our previous report found that most of the literature on procedures for extracting pharmaceuticals from the soil does not provide information on soil type and characteristics (Stando et al. 2022). It can be assumed that the soil composition (in particular, OC content) influences the recovery of the analytes. Understanding the relationship between soil matrix composition and analyte recovery is extremely important for accurate monitoring studies of soils with various compositions. Proper sample preparation is crucial, especially when determining trace amounts of compounds like SAs. The liquid chromatography coupled with tandem mass spectrometry (LC-MS/MS) technique is the most commonly used method due to its high sensitivity and selectivity provided by the aforementioned detector (Dmitrienko et al. 2014).

This study aimed to develop a procedure for isolating and determining SAs in soils with different compositions. Eight SAs most often detected in the various compartments of the environment were selected for the study: SMX, SFM, SFD, sulfapyridine (SFP), sulfathiazole (SFT), sulfamerazine (SFR), sulfamethizole (SFH), and sulfisoxazole (SFX) (Łukaszewicz et al. 2017; Kokoszka et al. 2021; Osiński et al. 2022). In the first step, soils with various OC content, pH, and elemental composition were used to develop the extraction procedure. LC-MS/MS was used to determine the SAs (Kokoszka et al. 2021). The procedure has been validated, and the recovery has been determined at four levels of OC content. The developed procedure was applied to environmental samples of soil collected in the Silesian Voivodeship. In monitoring studies, 27 soil samples were collected from areas where increased animal activity was observed. An additional aim of the study was to identify the TPs of SAs in soil.

## Materials and methods

### Chemicals and analytical standard solutions preparation

Analytical standards: SFD, SFR, SFM, SFH, SMX, SFP, SFT, and SFX, as well as chemical substances used in the sample preparation step, such as citric acid monohydrate, disodium phosphate dihydrate, and disodium EDTA dihydrate, were obtained from Sigma-Aldrich (St. Louis, MO, USA). Standards of Al, Ca, Mg, Na, K, solvents for ICP analysis (Suprapur^®^ 30% hydrochloric acid and Suprapur^®^ 65% nitric acid), and HPLC grade solvents used in LC-MS/MS analysis – water, 85% formic acid, methanol, and acetonitrile – were purchased from Merck (Darmstadt, Germany). Solvents (all analytical grade) used for extractions, (acetonitrile, methanol, 96% sulfuric acid, and formic acid) and substances used for soil characterization (anhydrous glucose and potassium dichromate) were obtained from Chempur (Piekary Śląskie, Poland). Analytical grade acetic acid was purchased from POCH S.A. (Gliwice, Poland), while technical grade pressurized nitrogen was bought from Siad (Ruda Śląska, Poland).

Lipophilic-hydrophilic balance (Oasis HLB 500 mg, 6 mL) and strong mixed-mode anion-exchange cartridges (Oasis MAX 150 mg, 6 mL) were purchased from Waters Corporation (Milford, MA, USA) and used for SPE extraction.

For each SAs, the solubility in different solvents was examined, and the solvent for each SA was selected experimentally based on its observed solubility. Five out of eight SAs standard solutions were prepared in methanol, SFP and SFR were dissolved in acidified methanol, and SFD in acetone. Stock solutions were prepared with concentrations of 1 mg mL^-1^. All the working stock solutions (mixtures of selected SAs) were obtained by diluting standard solutions with methanol.

### Sampling

Twenty-seven environmental surface soil samples (0–20 cm) were collected from cities in the Silesian Voivodeship, Poland, located in the temperate climate zone. From each sampling site, 1 kg of soil was collected from enclosed dog paddocks, recreational areas near the lakes, and agricultural fields. No information is available on fertilization of farmlands. “Envelope” sampling method was used and according to it from each sampling site five samples (one from each corner and one from the center of square area) were taken and mixed (Kapanadze et al. 2019). Then samples were homogenized and placed in plastic containers. Collected samples were immediately transported to the laboratory and stored at -70 °C before lyophilization (with average processing times as follows: 72 h at 0.100 mbar and -56 °C followed by 6 h at 0.028 mbar and -42 °C). The samples were then lyophilized and stored at 4 °C prior to analysis.

### Sample pretreatment

Two extraction techniques – SLE and SPE – were used for analytes extraction and sample enrichment. The entire method development was conducted on soil with medium OC content (blank sample without SAs) spiked with SAs standard stock solution to achieve the concentration of 500 ng g^-1^. The developed SLE-SPE procedure was additionally performed on arenaceous quartz (blank sample without SAs) with very low OC content for comparative purposes (Fig. S1).

Seven SLE procedures for isolating SAs from soil were examined (Table S1). SLE parameters, such as extraction solvents, shaking time, and the use of ultrasound, were tested to improve SAs recovery. The volume of the SLE solvent was constant at 10 mL for 1 g of soil.

The order of the performed steps was as follows – sonication (10 min), followed by shaking (30 or 60 min), and finally centrifugation (10 min). In procedures 2–7, extractions were repeated with new portions of solvent. An ultrasonic bath (USC 500 TH, Avantor, Radnor, Pa, USA) was used for sample sonification (all procedures except SLE3 and SLE7). Shaking was conducted with a Vibramax 100 orbital shaker with nine flask clamps (Heidolph Instruments, Schwabach, Germany) with 750 rpm selected as an optimum speed. A Hermle Z 323K centrifuge with a rotor for 12 tubes (Hermle Labortechnik, Wehingen, Germany) was used for isolating solid particles from the extracts. Solutions collected from each extraction were combined and diluted to 250 mL with water. The SLE step of the final developed SLE-SPE extraction procedure (SLE7+SPE8) involved adding 10 mL of methanol, acetonitrile, 0.1 M EDTA, and McIlvaine buffer pH=4 (30:20:25:25; V/V/V/V) to 1 g of soil. Then the soil-solvent suspension was shaken for 60 min at 750 rpm and centrifuged for 10 min at 8000 rpm. This step was repeated with another 10 mL portion of the extraction solvent.

Diluted extracts from the SLE, after adjusting the pH, were used for SPE. Eight SPE procedures (Table S2) with varied parameters, such as the SPE sorbent, pH, type and volume of conditioning and elution solvents, and the manner of eluate concentration before LC-MS/MS analysis, were tested.

An SPE apparatus with a 12-port vacuum manifold (BAKER spe 12G, Avantor, Radnor, Pa, USA) was connected to a vacuum pump (Rocker 400, Rocker Scientific, Kaohsiung, Taiwan) to obtain an optimum flow rate. Before the sample was passed through, the sorbent was conditioned with solvents in an appropriate order. For procedures SPE1–3 it was 6 mL of methanol, 6 mL 0.1 M HCl, and 6 mL H_2_O, while it was 6 mL of methanol, 6 mL H_2_O, and 6 mL McIlvaine buffer pH=4 for procedures SPE4–SPE8. As an eluant, methanol (SPE1–SPE6) or methanol acidified with fluoric acid or acetic acid (SPE7, SPE8) was applied. The eluate was dried under a stream of nitrogen and reconstituted with 1 mL of methanol (SPE1–SPE4). In procedure SPE6, following reconstitution the containers were additionally washed with MeOH:H_2_O (2:3; V/V) mixture. Another examined option was to partially dry the eluate to around 1 mL under a stream of nitrogen (SPE6–SPE8). MAX and HLB cartridges combined in tandem (SPE3) were conditioned together, the elution being conducted from the HLB cartridge after removing the MAX cartridge.

In the developed procedure prior to SPE, the sample pH was adjusted to 4 and an Oasis HLB cartridge was conditioned with 6 mL methanol, 6 mL H_2_O, and 6 mL McIlvaine buffer pH=4. The sample was loaded to the sorbent, which was then dried for 30 min, and the analytes were eluted with 12 mL 0.1% acetic acid in methanol. The eluate was dried under a stream of nitrogen to around 1 mL and then submitted for LC-MS/MS analysis.

### LC-MS/MS analysis conditions

SAs analyses were performed with a Dionex UltiMate 3000 HPLC system (Thermo Fisher Scientific, Waltham, MA, USA) coupled with an AB SCIEX 4000 QTRAP (Applied Biosystems/MDS SCIEX, Framingham, MA, USA) hybrid triple quadrupole-linear ion trap mass spectrometer as a detector. The LC system was equipped with an UltiMate 3000 autosampler, an UltiMate 3000 RS pump, and an UltiMate 3000 thermostated column compartment. A Kinetex F5 (Phenomenex, Torrance, CA, USA) core-shell column (100 × 2.1 mm; 1.7 μm) was employed for analytes separation at 25 °C with an injection volume of 2 μL. The mobile phase consisted of (A) 0.1% FA in H_2_O and (B) ACN, its total flow rate being 0.3 mL min^-1^. Elution was performed using the following gradient system: 0.0 min 90% A, 10% B; 3.0 min 80% A, 20% B; 7.0 min 40% A, 60% B; from 7.1 min the initial solvent composition (90% A, 10% B) was achieved with a total run-time of 10 min.

The detector was equipped with an electrospray ionization (ESI) source, which was operated in positive ion mode for all the SAs. For qualitative and quantitative analysis, multiple reaction monitoring (MRM) mode was used. The optimum ion source parameters were as follows: temperature, 500 °C; ion spray voltage, 4000 V; curtain gas, 20 psi; ion source gas, 55 psi; ion source gas 2, 55 psi. The optimized MS/MS parameters chosen for each analyte are listed in Table S3. They consisted of precursor ions (Q1), product ions (Q3), declustering potential (DP), collision energy (CE), collision cell exit potential (CXP), and entrance potential (EP).

### Soil characterization

Due to rich soil matrices and their significant impact on analytes determination, the OC content and pH of every soil used in this study was examined. The content of elements such as Al, Ca, Mg, Na, and K was also assessed because of scientific reports suggesting the possible effect of these ions on the adsorption of SAs in soils (Xu et al. 2021). The OC content was evaluated with an HP 8452A (Hewlett Packard, Palo Alto, CA, USA) UV-Vis spectrophotometer equipped with a DAD detector according to the PN-ISO 14235:2003 standard. Each soil sample was prepared in three replicates, each of which was measured three times. The percentage carbon content quantification was based on a calibration curve, and standard deviations were also calculated. The pH value was measured according to the ISO 10390:2021 standard using soil-water suspensions. For each sample, the soil measurement was performed three times.

Al, Ca, Mg, Na, and K were chosen due to their essential functions in the sorption of chemicals in soil. The exchange capacities of the cations of the abovementioned elements characterize a soil’s capability to adsorb other cations by exchange. The content of the selected elements was assessed using inductively coupled plasma atomic emission spectroscopy (ICP-AES). Initially, samples were prepared by microwave mineralization in aqua regia solution. To 0.5 g of lyophilized soil high purity acids (2.5 mL HNO_3_ and 7.5 mL HCl) were added. The sample was heated in a microwave mineralizer (Mars 5, CEM, Matthews, NC, USA) for 15 min at 200 °C and 1600 W. Prior to analysis, the samples were diluted with distilled water. A Varian 710-ES ICP-AES (Varian, Palo Alto, CA, USA) spectrometer was employed for the determination of elemental content; it had the following working parameters: RF power, 1.0 kW; plasma flow, 16 L min^-1^; auxiliary flow 1.5 L min^-1^; nebulizer pressure, 200 kPa; pump rate, 15 rpm. The emission lines were as follows: Al – λ=394.401 nm, λ=396.152 nm; Ca – λ=317.933 nm, λ=370.602 nm, λ=422.673 nm; Mg – λ=279.800 nm, λ=280.270 nm; Na – λ=588.995 nm, λ=589.592 nm; K – λ=766.491 nm, λ=769.897 nm. Samples were prepared in three replicates, each of which was measured three times. A calibration curve was used for calibration, and the results were calculated as an average value obtained for all analytical lines of the selected element.

Correlation analysis was conducted on data from nine different soils (SWI, SWIII, SWIV, SWV, SPI, JK2, PM, WLII, and a blank soil sample used as a model) using Statistica 13 software (TIBCO Software Inc., Palo Alto, CA, USA). The soil samples were enriched with SAs prior to extraction, resulting in a final yield of 500 ng g^-1^ for each sample (three repetitions for each soil). Initial concentrations of selected SAs in the soils (before spiking) were measured to determine the precise amount of standard solution required for the enrichment to obtain final concentration 500 ng g^-1^.

### Method validation

Validation of the developed SLE-SPE-LC-MS/MS method for analysis of SAs was conducted. Parameters such as accuracy, precision, linearity, limit of detection (LOD), limit of quantification (LOQ), matrix effect (ME), and recovery were determined. The soil matrix extract (medium amount of OC and neutral pH) prepared according to the procedure described in Method validation (Results and discussion section) was used to determine the validation parameters. Due to the presence of trace amounts of the determined SAs in the selected soil, a standard addition method was performed concerning blank sample. Calibration curves were constructed over the concentration range 1–500 ng L^-1^ by adding the appropriate volume of working standard solutions to extracts from soil with a medium OC content and neutral pH (soil sample SWIV). These measurements were also used for linearity and coefficient of determination (R^2^) calculations for all analytes. The LOD values were obtained from equation (1):

1 $$LOD=\frac{LOQ}{3}$$

The LOQ of each SA was the calibration curve’s lowest values for which the precision and accuracy were lower than 20%. For ME evaluation, samples were prepared by adding SAs standards mixture to both 0.1% AcA in MeOH and soil extract. The ME was calculated from equation (2).

2 $$ME=\frac{\left(A-B\right)}{A}\cdot 100\%$$

Where:

A: area under the peak for analyte in 0.1% AcA in MeOH
B: area under the peak for analyte in matrix soil extract

Quality control (QC) samples were made up at three concentration levels – low (LQC=10 ng g^-1^), medium (MQC=250 ng g^-1^), and high (HQC=400 ng g^-1^) – by adding the appropriate volume of working standard solutions of selected SAs to matrix extracts from soil SWIV. QC were used to assess accuracy (relative error – RE), precision (coefficient of variation – CV), and recovery (R) of selected SAs at four level ranges of OC content. R was calculated from equation (3).

3 $$R=\frac{x_2}{x_1}\times 100\%$$

Where:

x_1_: amount of analyte in sample before pretreatment
x_2_: amount of analyte after sample pretreatment

### Identification of SAs transformation products in soil samples

LC-MS/MS operated in various modes was also used for the identification of TPs in soil extracts. The chromatographic separation conditions and ion source parameters were the same as for the targeted analysis. TPs were detected and identified in a few steps using the QTRAP mass analyzer’s various operation modes. In the first step, a screening analysis was performed using the pseudo-multiple reaction monitoring (p-MRM) mode. The p-MRM method was built with the LightSight^TM^ software, and a SAs TPs data set was constructed based on the literature. The p-MRM mode has been successfully used previously in the screening analysis of SAs TPs in water samples (Kokoszka et al. 2021). Then, QTRAP linear ion trap scan modes, such as the enhanced mass scan (EMS) and enhanced product ion scan (EPI) modes, were used for non-target analysis.

The information-dependent acquisition (IDA) mode, combining EMS with EPI, was used to maximize the information obtained in one scan. Data recorded by the EMS-IDA-EPI method were collected in positive (ESI+) and negative (ESI-) ionization modes, while the p-MRM method data were collected only in positive ionization mode. The EMS and EPI mass ranges were from m/z 50 to m/z 700, and the scan rates were 1000 Da s^-1^. The IDA criteria were as follows: the trigger for EPI was the 1–2 most intense ions that exceeded 100 cps; the mass tolerance was 250 mDa; former target ions were excluded for 30 s after two occurrences; the maximum rolling collision energy allowed was 80 eV in ESI+ and -80 eV in ESI-; and the dynamic background subtraction was turned on.

The presence of TPs in the soil extracts identified by the p-MRM mode was confirmed by analyzing the mass spectra recorded in the EMS-IDA-EPI mode. In the last step, mass spectra (ESI+ and ESI-) of TPs that most likely derived from SAs were selected. Non-targeted analysis was performed using a retrospective approach to mass spectral analysis. The TPs identified in this way were compared with information from databases or the literature if possible.

## Results and discussion

### Development of a method for extracting SAs from soils with distinct characteristics

**Fig. 1:**
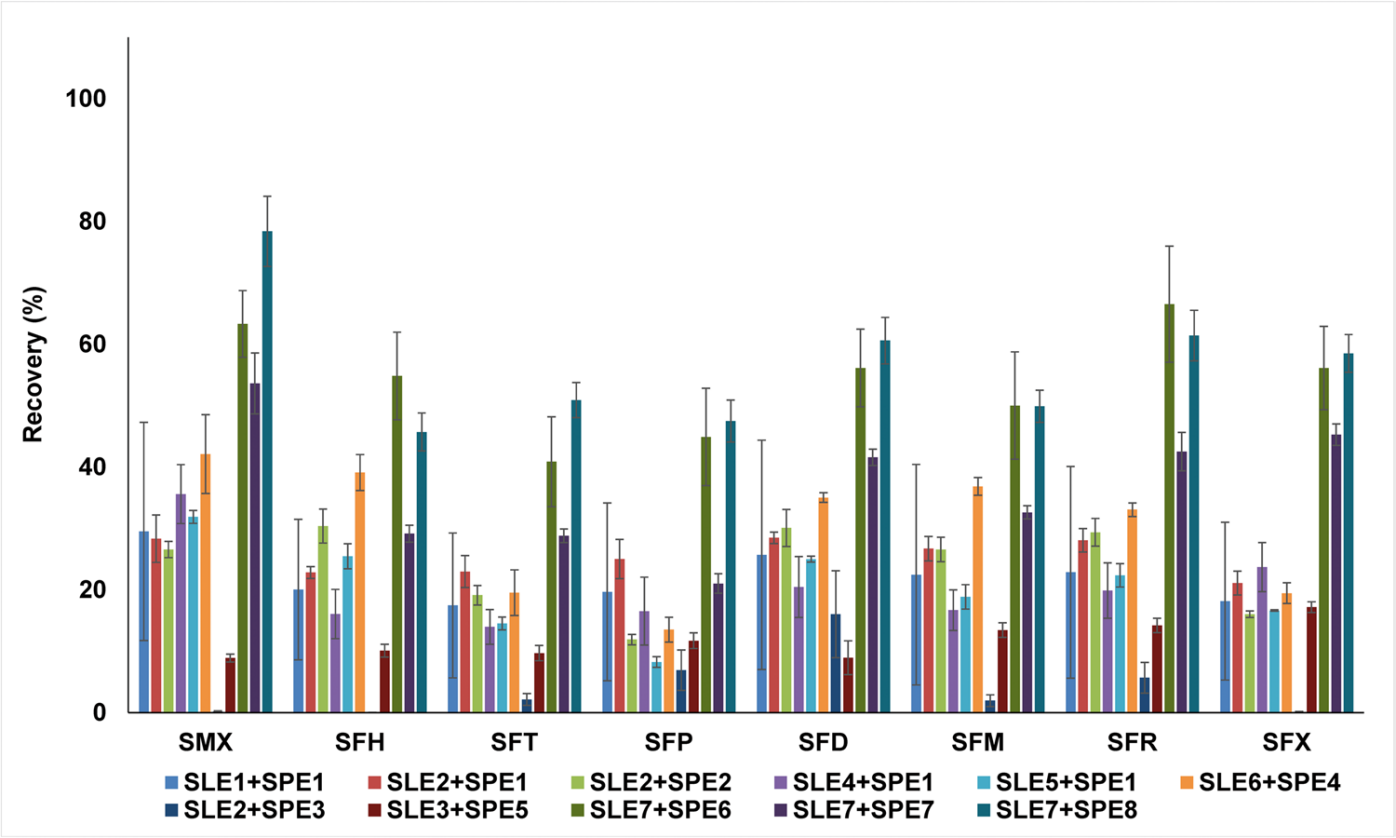
Recoveries obtained for 11 SLE-SPE procedures for SAs extraction from blank sample used as model soil

Complex matrices specific to soil samples may cause difficulties in the quantitative assessment of selected pharmaceuticals using electrospray ionization due to the matrix effect (Taylor 2005; Rossmann et al. 2015). It is crucial to apply solutions that will compensate for the impact of co-eluted compounds. To develop the universal SLE-SPE procedure for a different types of soils, the following parameters were investigated: SLE solvents, use of ultrasound, shaking time, and for SPE – sample pH, a tandem combination of cartridges, conditioning solvents, elution solvents, and eluate preparation before LC-MS/MS analysis. The recoveries obtained for the 11 SLE-SPE procedures are summarized in Table S4 and shown in Fig. 1.

Our previous study showed that SAs were more stable in McIlvaine buffer than in citrate buffer (Stando et al. 2022). The effectiveness of dual extraction was also examined. A mixture of McIlvaine buffer (pH=4) and acetonitrile (1:1; V/V) (SLE2) provided better repeatability with overall higher recoveries for the same SPE procedure (SPE1), but all in a low range (21.1–28.5%). Procedures with an increased shaking time and without sonication (SLE3), with the addition of an ion exchanger (sodium fluoride – SLE4) (Hou et al. 2015), and with separate extractions with aqueous and organic solvents (SLE5) were also examined (Table S1). None of the abovementioned modifications had a relevant influence on recovery increase (all below 35.6% for each SA). However, the use of both water and of organic solvents mixture – MeOH, ACN, 0.1 M EDTA, and McIlvaine buffer (pH=4) (30:20:25:25; V/V/V/V) – allowed satisfactory results to be obtained (SLE6 – up to 42.1%), better for the procedure without ultrasound and with shaking for 60 min (SLE7). In the best SLE-SPE combination, the recoveries increased for each SA. The addition of the chelating agent EDTA impacted positively on extraction effectiveness by showing an ability to bind metal ions, e.g., Ca^2+^ and Mg^2+^, that facilitate SAs adsorption to soil particles (Carmona et al. 2017; Xu et al. 2021).

A SPE procedure for extraction of the target SAs from water samples was published previously (Kokoszka et al. 2021) and was used here as an initial procedure (SPE1). SAs have varied pK_a_ values, but generally, they have a cationic form at a pH below 3 and an anionic form above 4.5 (Baran et al. 2011). While developing an extraction procedure for liquid samples, it was proven that a pH in the range 3.0–4.5 was beneficial, so the SLE2+SPE1 procedure was additionally performed with a sample of pH=3 (SLE2+SPE2). The recoveries were similar, scarcely lower, but the repeatability for each SA decreased, so pH=4 was chosen as an optimum value. The anion-exchange sorbent (Oasis MAX) in tandem with an Oasis HLB cartridge (SLE2+SPE3) was used for preliminary sample cleaning from matrix components (Hu et al. 2012). Nevertheless, the obtained recoveries were significantly lower (lower than 16.0%). A possible explanation is too strong adsorption of analytes to the MAX sorbent, which can interact with the cationic form of SAs. Eventually, Oasis HLB cartridges alone were used for SPE.

Initial conditioning solvents for selected SAs were 6 mL methanol, 6 mL 0.1 M HCl, and 6 mL H_2_O. This combination gave satisfactory results for liquid samples, but insufficient values for soils (SPE1-SPE3) when combined with SLE-SPE procedures. However, using 6 mL methanol, 6 mL H_2_O, and 6 mL McIlvaine buffer (pH=4) as sorbent conditioning solvents (SPE4-SPE8) significantly increased the recoveries for all target compounds (recovery in the range 19.5–42.1%) (Ho et al. 2012). One of the principal factors that demonstrated an impact on extraction effectiveness was eluate pretreatment before LC-MS/MS analysis. The first option was to dry to dryness under a stream of nitrogen and dissolve the residues in 1 mL of methanol. Another set of conditions was to additionally wash the vials after carrying the sample with methanol and water (2:3; V/V) mixture (SLE3+SPE5). This additional wash did not improve extraction efficacy. The best results were obtained after dispensing with the concept of complete drying. Instead, the eluate was evaporated to around 1 mL and analyzed (SPE6-SPE8). SAs can adsorb onto glass walls in small amounts (Shikuku et al. 2018), and this effect was partially eliminated by limited evaporation. Comparing procedures SLE7+SPE6 and SLE7+SPE7, it was noticeable that the addition of FA in MeOH as the elution solvent reduced SAs recoveries compared to MeOH alone, as follows: 21.0–53.6% (FA in MeOH) and 40.9–66.5% (MeOH). The use of acetic acid in MeOH as the eluent gave better or comparable results to MeOH (45.7–78.4%) and was therefore used in the final procedure. This effect can be caused by the strength of the acids employed. Acetic acid is weaker and decreases pH to a lesser extent than formic acid, to a value that may have a favorable impact on the elution process of the selected SAs. The final developed SLE-SPE procedure for target analytes extraction from soils was SLE7+SPE8 (Fig. 1), which had the highest recoveries and acceptable repeatability for all analytes. While the two-stage extraction procedure could be seen as a potential drawback, its strength lies in its adaptability to various soil types.

To obtain an effective and universal procedure for soils with distinct characteristics, the influence of OC content on SAs recovery was determined. Furthermore, pH and elemental content (Al, Na, K, Ca, Mg) was determined for expanded soil characterization. Detailed information on the 27 collected soil samples and their characteristics are presented in Table S5.

The OC content was assessed for 27 environmental soil samples. These were divided into three groups, giving 6 soils with very low, 4 with low, and 17 with medium OC content based on European Soil Bureau guidance. Following our previous research, it was assumed that the increase in organic matter content was responsible for the lower recoveries for the same extraction procedure (Stando et al. 2022). Therefore, the extraction effectiveness of the final SLE-SPE procedure was compared for arenaceous quartz (very low OC content – below LOQ) and a blank sample used as a model sample (medium OC content – 3.02%) (Fig. S1). It was clear that recoveries significantly rose for arenaceous quartz where the matrix was deprived of organic matter, which probably caused stronger SAs adsorption in model soil (Li et al. 2011; Ho et al. 2014). The pH value was evaluated for each soil, which were then divided into five groups according to following criteria: very acidic (pH<5.0), acidic (pH 5.1–6.0), slightly acidic (pH 6.1–6.7), neutral (pH 6.8–7.4), and basic (pH>7.4) (Mocek 2020). Considering the obtained results, the following number of soils were assigned to each group: 3 acidic, 6 slightly acidic, 7 neutral, and 11 basic. This gave a large diversity of samples in terms of this parameter. Another important consideration was that as the pH of the soil rose, the cation exchange capacity increased as well. This phenomenon is caused by an increase in the negative charge of organic and mineral substances because of the deprotonation of functional groups (Shuey 1990).

To confirm the statistical influence of organic carbon content on recovery, Spearman's rank correlation coefficients were calculated. The results were as follows, sequentially: -0.7167, -0.8833, -0.9000, -0.8667, -0.8167, -0.8833, -0.8500, -0.8667 for SMX, SFH, SFT, SFP, SFD, SFM, SFR, and SFX. All of these results were statistically significant (p<0.05). A strong negative correlation between recovery and OC was shown for each of the SAs, which confirms that as the OC content increases, the SAs recovery decreases.

### Method validation

The developed UHPLC-MS/MS method was validated, and the validation results are presented in Table 1. Parameters such as linearity, LOD, LOQ, accuracy, precision, ME, and recovery were determined. Soil sample extracts with a medium OC content were used for validation, and nine independent repetitions were performed each time. The calibration curves for SAs were linear from 1.0 to 500.0 ng g^-1^. The R^2^ was in the range of 0.9890–0.9959. The LOD and LOQ for all SAs were 0.33 and 1.0 ng g^-1^, respectively. Accuracy and precision were determined based on the analysis of QC samples at three concentration levels: 10 ng g^-1^ (low), 250 ng g^-1^ (medium), and 400 ng g^-1^ (high). Precision, defined as the CV for all SAs, was less than 15%. Accuracy determined by the RE was in the range of -31% to 30%. For SMX, SFH, SFM, and SFX, the ME was negligible (ME: -3.0–13%), while for SFR, SFP, SFT, and SFD, ion enhancement was observed in the 17–36% range. The method’s selectivity was achieved using the MRM MS/MS operating mode. No interference peaks were observed at the retention times of the selected SAs in blank soil extract samples.

**Table 1:** The analytical parameters of the developed SLE-SPE-UPLC-MS/MS procedure

Analyte	Linear range (ng g^-1^)	R^2,a^	LOD^b^ (ng g^-1^)	LOQ^c^ (ng g^-1^)	Concentration (ng g^-1^)	CV(%)^d^	RE(%)^e^	ME^f^ (%)	Recovery±SD^h^ (%)			
									OC<1 (%)^g^	1 ≤OC<2 (%)	2≤OC<3 (%)	3 ≤OC (%)
SMX	1-500	0.9893	0.33	1.0	10	7.2	18	-6.4	110±14.8	71.6±4.1	69.6±6.6	50.4±5.1
					250	4.2	-3.5		98.3±12.2	78.0±7.6	69.4±3.5	43.2±8.7
					400	1.5	-0.4		99.1±10.6	80.5±2.5	65.5±8.1	50.5±7.6
SFH	1-500	0.9959	0.33	1.0	10	5.2	-33	1.6	90.3±12.8	42.6±5.0	42.3±1.9	28.7±3.6
					250	7.7	-3.3		88.8±10.8	42.9±6.3	50.8±4.6	20.8±2.3
					400	2.8	-4.4		83.1±12.6	57.6±3.9	50.1±6.0	29.0±3.4
SFT	1-500	0.9902	0.33	1.0	10	6.7	-18	18.0	83.1±7.6	45.5±3.8	31.6±2.8	31.5±2.3
					250	7.1	-0.1		89.0±10.3	41.5±2.6	33.1±4.1	23.6±2.3
					400	9.5	0.7		88.7±10.9	56.4±1.6	41.2±5.5	28.0±2.8
SFP	1-500	0.9894	0.33	1.0	10	15	2.8	17.0	105±8.7	53.4±6.3	37.5±4.1	38.1±4.6
					250	6.7	3.7		88.0±3.4	43.5±5.8	39.6±4.8	33.9±4.0
					400	7.1	-11		95.8±15.3	65.5±7.1	56.1±7.4	41.2±5.1
SFD	1-500	0.9910	0.33	1.0	10	8.6	30	36.0	91.0±8.1	74.1±8.3	53.8±6.8	37.6±5.8
					250	3.7	-3.0		96.9±4.6	63.3±4.7	58.6±7.1	30.7±3.2
					400	0.3	2.4		100±13.7	74.9±5.5	69.9±9.7	50.8±4.7
SFM	1-500	0.9890	0.33	1.0	10	10	-31	13.0	109±8.9	49.0±3.0	49.0±5.0	24.4±3.4
					250	7.3	2.8		95.4±9.3	62.4±7.8	55.4±6.3	27.8±2.8
					400	2.8	5.5		99.3±14.7	68.4±3.0	57.2±7.8	40.2±5.4
SFR	1-500	0.9943	0.33	1.0	10	9.1	-21	19.0	106±13.0	48.0±7.0	52.1±2.1	32.5±4.1
					250	6.3	-1.4		103±7.1	50.8±2.9	42.7±4.5	29.7±3.1
					400	2.5	0.3		100±13.1	68.7±4.2	60.6±8.2	45.3±4.9
SFX	1-500	0.9901	0.33	1.0	10	14	-19	-3.0	81.6±7.4	67.7±8.4	58.8±5.7	42.9±2.6
					250	5.8	-5.8		81.6±11.9	67.8±2.6	51.0±6.3	41.5±4.9
					400	7.1	-3.2		82.0±9.8	69.0±3.6	56.8±.7.7	41.6±3.1

^a^R^2^ – coefficient of determination; ^b^ LOD – limit of detection; ^c^ LOQ – limit of quantification; ^d^ CV – coefficient of variation; ^e^ RE – relative error; ^f^ ME – matrix effect; ^g^ OC – organic carbon content; ^h^ SD – standard deviation

Number of repetitions for calculations: n=9

To develop an extraction method suitable for environmental monitoring studies, the validation was extended to the determination of recoveries for soils with various OC contents. Extracts of samples of these soils (very low to medium OC content) were analyzed with the addition of appropriate amounts of SAs. Based on the guidelines of the European Soil Bureau, the OC content was classified according to the following criteria: very low: OC<1.0%, low: 1.0-2.0%, medium: 2.0-6.0%, high: OC>6.0% (Gonet 2007). The adopted range of OC content in the medium criterion was too wide to assess the impact of OC on recovery objectively. For this reason, this study distinguished between medium-low (2≤OC<3) and medium-high (3≤OC) ranges. The obtained recoveries differed depending on the OC content in the soil. A decrease in SAs recoveries is related to an increase in the OC content of the soil. The SAs recoveries for soils with very low, low, medium-low, and medium-high OC content changed by 81.6–110%, 41.5–80.5%, 31.6–69.9%, and 20.8–50.8%, respectively. According to the literature, SAs sorption in soil increases with OC content, which may result in low recovery of analytes (Leal et al. 2013). Another factor reducing the SAs recovery is the coextraction of matrix compounds with analytes in SLE. In our previous study, we showed that the presence of soil matrix components significantly reduces the recovery of pharmaceuticals at the SPE stage (Stando et al. 2022). Therefore, we recalculated the concentrations of quantified SAs based on the aforementioned four recovery ranges established for various OC contents.

The developed SLE-SPE-LC-MS/MS method was effective, accurate, and precise. According to the literature, the SAs content in various soil types is in the 0.04–500 mg kg^-1^ range (Cycoń et al. 2019). The established procedure is sufficiently sensitive to determine SAs residues in the environment. Determining recoveries at four levels of OC made it possible to obtain accurate results for soil samples with various compositions and confirmed the (Shelver et al. 2010; Ho et al. 2012; García-Galán et al. 2013) universality of the proposed SLE-SPE procedure. Despite the availability of many procedures for extracting SAs from the soil, their comparison with the newly developed procedure is difficult due to the lack of or incomplete information on the soil composition. To our best knowledge, this is the only study that links the effects of OC at various levels to SAs recovery obtained after extraction from soil samples.

### Detection of SAs residues in soil samples from areas with increased animal activity

Monitoring of SAs in soils was carried out in six cities in the Silesian Voivodeship – the most urbanized and industrialized area in Poland. Twenty-seven sampling points that met the criterion of increased activity of animals were selected. The areas with "increased animal activity" can be understood broadly and will refer to the various areas, depending on urban, suburban, and agricultural areas. The determined concentrations of SAs in soil samples are listed in Table 2. The main route of SAs introduction to the soil is livestock manure, which is used as a natural fertilizer (Zhao et al. 2019). However, not only agricultural area is exposed to the accumulation of SAs. The same compounds are used in the treatment of farm animals (calves, castles, swine, horses) and in the treatment of domestic animals (mainly cats and dogs) (Hsu 2008). In both farm and domestic animals’ treatment, SMX (Bactrim^®^), SFD (Tribrissen^®^), and Sulfadimethoxine (Albon^®^) are used. SFM (Sulmet^®^) and Sulfasalazine (Azulfidine) are also used to treat dogs and cats (Hsu 2008). In urban areas, places with increased activity of animals, and thus the most exposed to SAs accumulation are parks, dog and horse paddocks, separate playgrounds for pets, green regions in the vicinity of residential estates together with tourist centers, due to the many domestic animals present.

**Table 2:** Concentrations of selected SAs in soil samples collected from areas with increased animal activity

Abbreviation	SAs concentration (SD) (ng g^-1^)							
	SMX	SFH	SFT	SFP	SFD	SFM	SFR	SFX
CPDG	-	-	-	-	-	-	-	-
PPDG	<LOQ	<LOQ	<LOQ	-	1.5 (0.5)	<LOQ	2.3 (0.5)	-
ŚPDG	-	-	-	-	-	-	-	-
PZDG	2.3 (1.0)	-	-	-	2.5 (0.5)	-	-	-
SBP	<LOQ	<LOQ	<LOQ	-	1.6 (0.5)	<LOQ	2.6 (0.7)	4.3 (1.0)
SBŚ	2.3 (0.1)	5.5 (1.3)	-	7.0 (0.3)	4.5 (0.2)	3.2 (0.3)	4.9 (1.3)	4.4 (0.6)
WLI	2.2 (0.3)	3.1 (0.7)	-	5.3 (0.6)	3.6 (0.7)	2.6 (0.5)	-	-
WLII	2.3 (0.4)	4.1 (0.4)	<LOQ	6.0 (0.6)	4.3 (0.5)	2.9 (0.3)	<LOQ	4.5 (0.4)
KBS	<LOQ	-	<LOQ	-	-	-	-	-
KBP	2.9 (0.3)	2.5 (0.7)	1.9 (0.3)	2.3 (0.6)	3.7 (0.8)	2.4 (0.7)	2.2 (0.4)	2.4 (0.5)
SKM	<LOQ	-	-	-	-	-	-	-
PM	<LOQ	-	-	-	<LOQ	-	-	-
PP	<LOQ	-	-	-	-	-	-	-
SWI	10.5 (1.0)	-	-	-	<LOQ	-	-	-
SWII	<LOQ	-	-	-	<LOQ	-	-	-
SWIII	1.9 (0.4)	6.5 (0.7)	2.2 (0.7)	7.6 (0.6)	6.3 (0.7)	2.7 (0.2)	<LOQ	6.0 (0.9)
SWIV	<LOQ	-	-	-	<LOQ	-	-	-
SWV	2.9 (0.4)	5.2 (1.0)	-	6.3 (1.4)	3.5 (0.4)	3.5 (0.5)	1.7 (0.2)	-
SPI	2.2 (0.2)	3.6 (0.3)	-	4.9 (0.8)	5.4 (0.8)	2.3 (0.3)	<LOQ	3.9 (0.7)
SPII	<LOQ	3.2 (0.4)	-	5.0 (0.9)	4.1 (0.6)	<LOQ	<LOQ	4.8 (0.3)
SWET	-	-	-	-	2.0 (0.4)	-	-	-
OSPP	<LOQ	<LOQ	-	<LOQ	2.0 (0.5)	<LOQ	2.2 (0.2)	7.1 (1.7)
JKI	-	-	-	-	-	-	-	-
JKII	<LOQ	-	-	-	-	-	-	-
PCENT	2.0 (0.2)	3.0 (0.6)	-	3.9 (0.6)	2.8 (0.2)	2.3 (0.3)	-	-
DPP	<LOQ	<LOQ	-	-	<LOQ	-	2.2 (0.2)	-
PN	<LOQ	-	-	-	-	-	-	-

“-“ – not detected; LOQ=1.0 ng g^-1^ for all SAs

Each of the selected eight SAs was determined in soils collected from dog paddocks. The SMX maximum concentration among samples from dog paddocks was 10.5 ng g^-1^ (SWI). Higher concentrations of SAs were found in samples taken from fenced dog paddocks (SWI-SWV), compared to non-fenced (SPI, SPII, SWET), which were respectively in the ranges 1.7–10.5 ng g^-1^ and 2.2–5.4 ng g^-1^. There were no significant differences in the concentrations of SAs collected from the paddocks in Katowice and Sosnowiec. In both cities, there was one dog paddock where all eight SAs were determined in the soil, in concentrations ranging from 2.3–6.0 ng g^-1^ (WLII, Katowice) and 1.9–7.6 ng g^-1^ (SWIII, Sosnowiec). Poland contains an estimated 6–8 million dogs and 37.78 million people, which means that statistically, every 5th person in Poland has a dog (Wierzbowska et al. 2016). It can be concluded that the factors influencing the increased accumulation of SAs in dog runs are the small usable area of the paddocks (300–2000 m^2^), the large population of dogs in cities, and the widespread use of drug therapy in domestic animals. SMX was detected in soils collected from a horse paddock in the city center of Sosnowiec (KBS) and from another in the suburban area of Mikołów (SKM), but their concentrations were below the LOQ. The low amount of SAs detected in soils from horse paddocks compared to dog paddocks is related to the specificity of the population of these animals. Specific breeders own horse paddocks, so the population of horses in a given area is significantly limited, and sick individuals are isolated from healthy ones. Dog runs are public places where the rotation of dogs during the day is high, and the only limitation is their space.

Agricultural fields are not directly exposed to animal activity, and the source of SAs contamination is animal manure used as fertilizer. As a part of the research, three fields in suburban areas (PM, PP, and KBP) where animal manure was used in the last two years were selected. The method of fertilizing the field by the owners and the type of manure used is unknown. Trace amounts (<LOQ) of SMX and SFD were detected in soils from PM and PP. All eight SAs were determined in the KBP soil in a concentration range of 1.9–3.7 ng g^-1^. The tendency to accumulate SAs in the soil after fertilization with manure has been the subject of many studies (Ho et al. 2012; Li et al. 2015; Zhao et al. 2019). The concentration of SAs in greenhouse soils and soils from open fields after fertilization with manure were respectively in the following ranges: 0.06–1.1 μg kg^-1^ and 0.04–0.1 μg kg^-1^ (Li et al. 2015). The SFD content in nine soils fertilized with manure was below 7 μg kg^-1^ (Ho et al. 2012). In soil fertilized over a long period of time with manure containing SMX, SFR, and SFM, the SAs were much lower (1.63–4.18 ng g^-1^) than for soil in which organic fertilizer was used (114.42–16,858.38 ng g^-1^) (Zhao et al. 2019). It is noticeable that the content of SAs obtained from PM, PP, and KBP soils was similar to the literature data. In addition, the concentration range in which SAs were detected in soil samples from the agricultural fields was lower than for soil samples taken from dog paddocks.

In samples of soil extracts collected at the Paprocany tourist center (OSPP, PCENT, and DPP), all the SAs were determined, except for SFT. OSPP and DPP were sandy samples with the lowest OC content (OC<LOQ) (Table S5). The concentration of SFX, SFR, and SFD in these samples was in a range of 2.0–7.1 ng g^-1^, while the remaining SAs (SMX, SFH, SFP, SFM) were below the LOQ. In the soil extract collected from the center of the tourist resort (PCENT), five SAs in a concentration range of 2.0–3.9 ng g^-1^ were detected. In Paprocany lake SMX and SFP were previously detected at concentrations of 75.88 ng L^-1^ and 22.99 ng L^-1^, respectively (Kokoszka et al. 2021). These results show that SAs are continuously introduced into the water and soil system in the Paprocany center. Soil samples taken from the Pogoria tourist center (CPDG, PPDG, and SPDG) had a low OC content (OC<LOQ). Only in extracts from PPDG were six SAs detected in a concentration below 2.3 ng g^-1^. The absence of SAs in CPDG and SPDG can be explained by the proximity of Pogoria Lake, which could wash out the SAs. SAs are easily leached from the soil, which allows them to migrate into the environment (Albero et al. 2018). All eight SAs were identified in soil extracts collected near Borki Lake (SBP, SBS). The concentration of SAs in SBP and SBS were in the ranges 1.6–4.3 ng g^-1^ and 2.3–7.0 ng g^-1^, respectively. We suspect that the presence of SAs in soil extracts may be related to the high activity of visiting domestic animals or the migration of pollutants in the environment associated with runoff from agricultural fields.

This study also considered green areas located in the Dąbrowa Górnicza (PZDG) and Tychy (JK1, JK2, and PN) city centers. The lowest levels of SAs compared to dog paddocks and agricultural fields were found in the green areas. In PZDG, SMX and SFD were detected at concentrations of 2.3 ng g^-1^ and 2.5 ng g^-1^, respectively. In PN and JK2, only SMX was detected, and its concentration was below the LOQ. In JK1, no SAs were detected. Literature data on the occurrence of SAs in soils in urban areas are limited. In the report of Xinzhu Yi et al., SAs residues were detected in soil and surface water samples from nine urban parks and five open spaces. SAs were detected at a higher concentration in groundwater (0.1–10 ng L^-1^) than in soils (<1 ng g^-1^) (Yi et al. 2019b).

All eight selected SAs were detected in soil samples collected in urban and suburban areas. The highest concentrations of SAs were found in dog runs, which confirms that increased activity of domestic animals in the areas designated for them results in the accumulation of SAs in the soil. The content of SAs in soils collected from dog runs (WLI, WLII, SWI, SWII, SWIII, SWIB, SWV, SPI, and SPII) was higher than for those collected from agricultural lands (KBP, PM, and PP). The most widespread SAs were SMX (23 places) and SFD (19 places). No SAs were detected in soils collected near water reservoirs (CPDG, SPDG, and JK1), which may be caused by washing out.

### Identification of transformation products of SAs in soil extracts

A total of 29 TPs of SAs were detected in 24 out of 27 extracts from soil samples. The degradation of SAs most often occurs by breaking the S-N, N-C, or C-S bonds between the SA core and the aromatic amine. In Table 3, the structures of the SAs TPs are presented, along with the mass of the molecular ion and fragment ions, while in Table S6 the distribution and frequency of the detected SAs TPs in soil samples are shown.

**Table 3:**
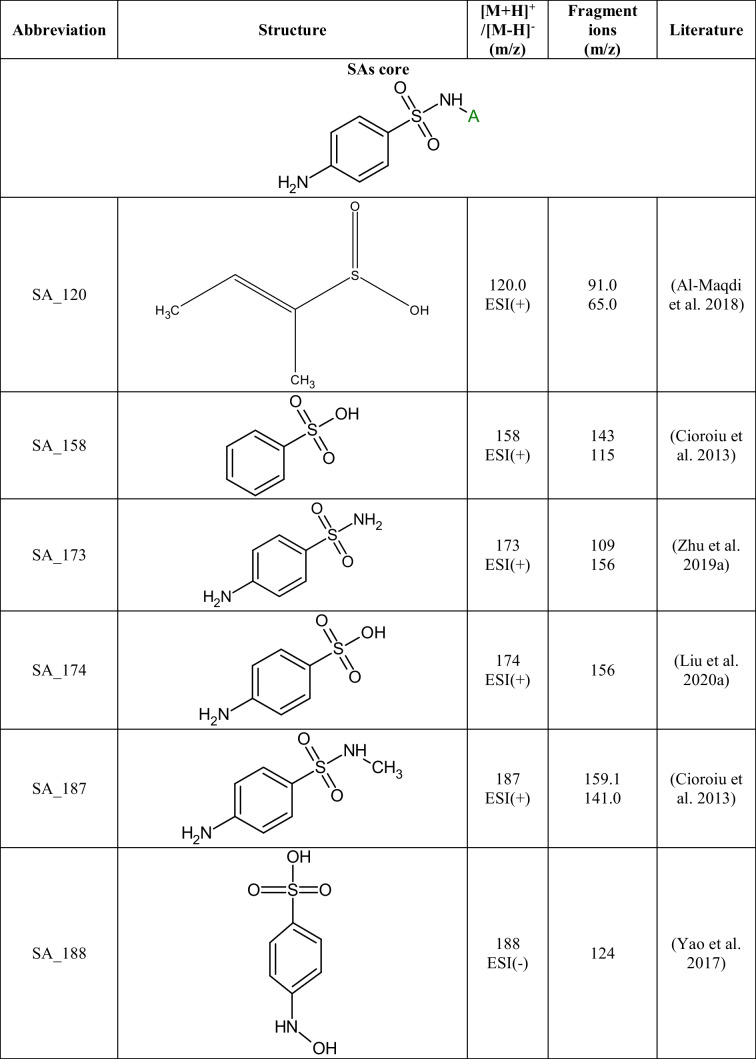

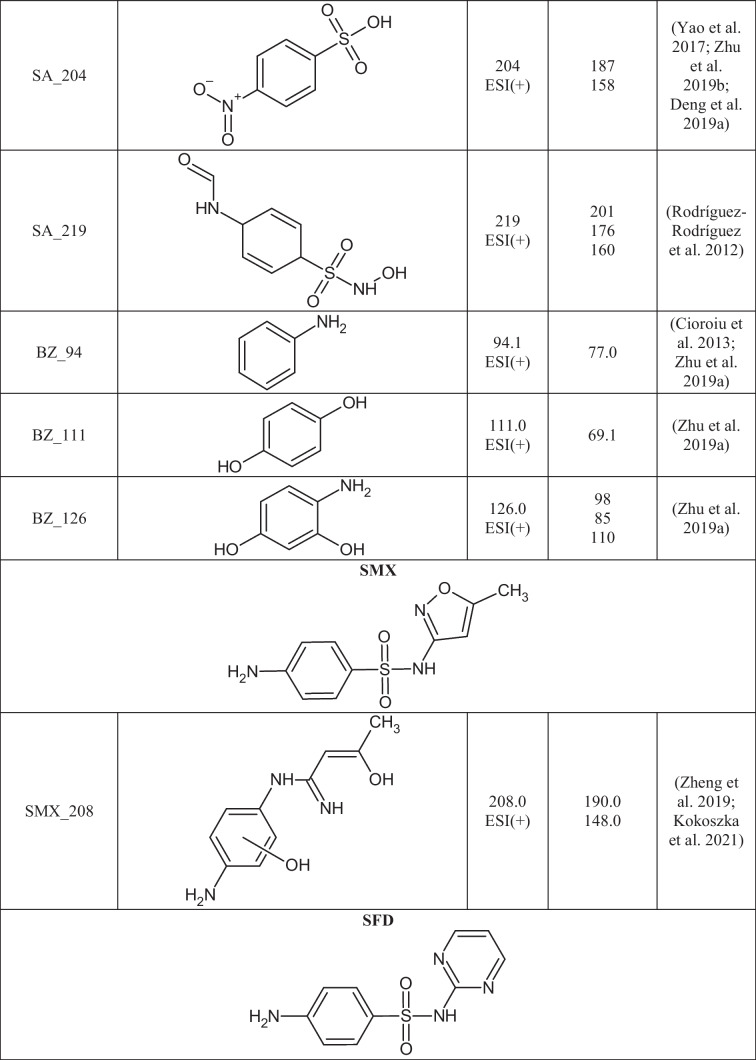

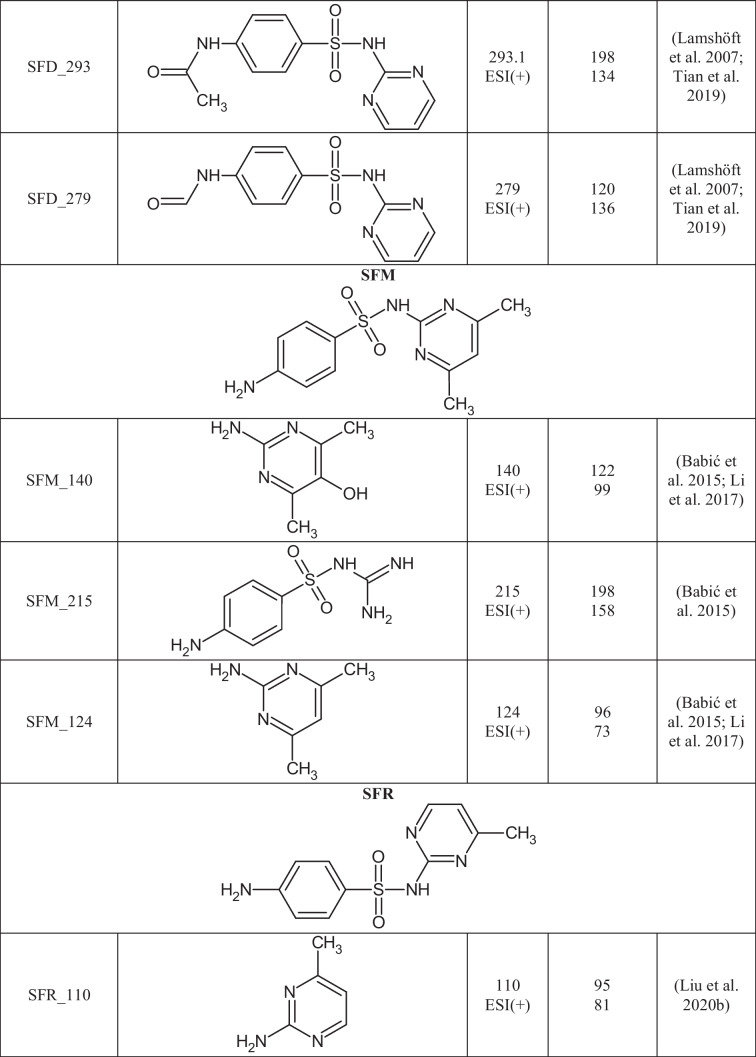

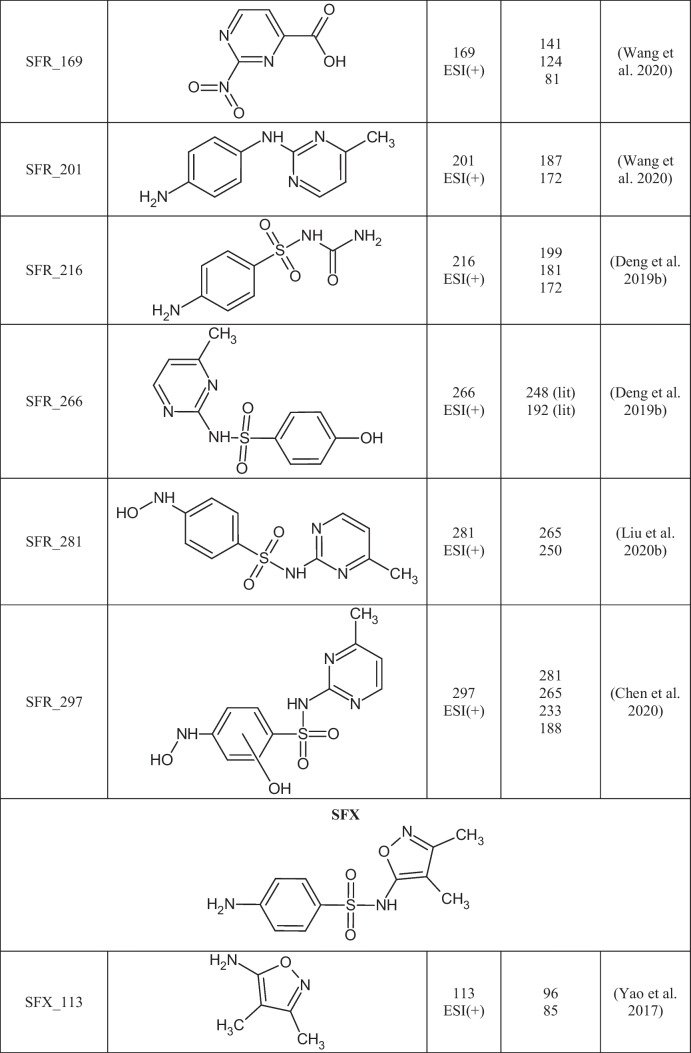

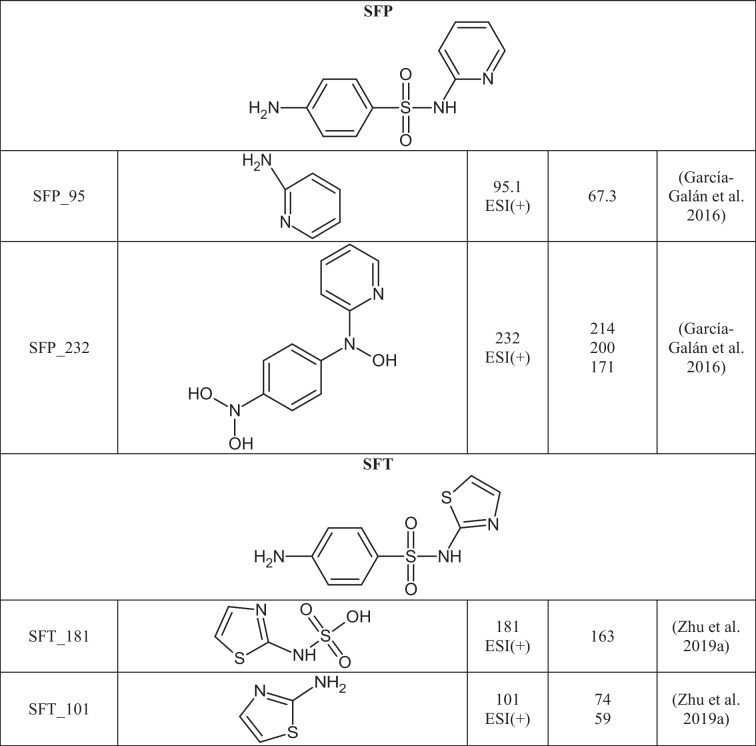
Structures of SAs transformation products detected in soils

**Fig. 2:**
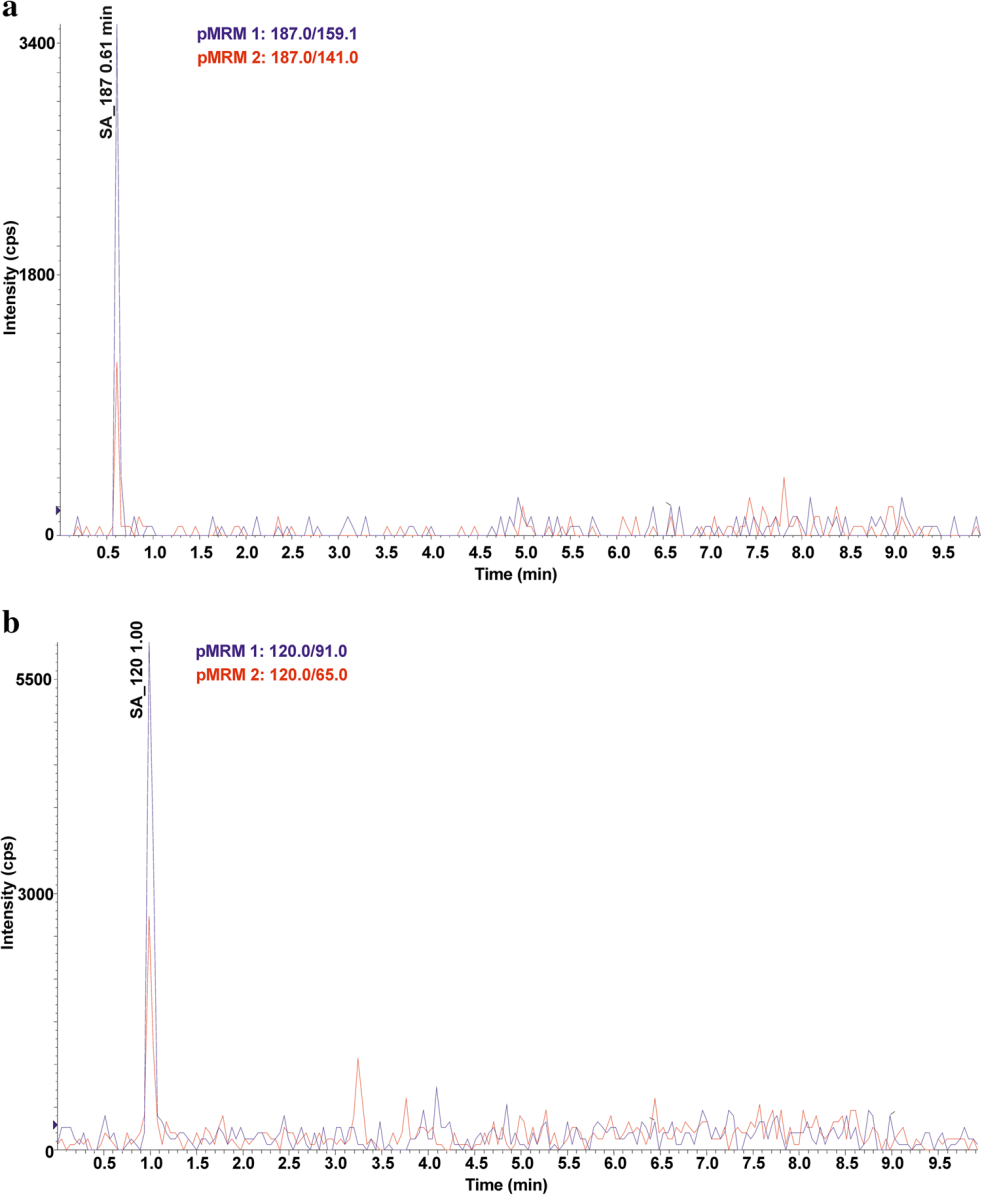
Chromatograms of the selected TPs obtained in p-MRM mode: a) SA_187 (m/z 180) and b) SA_120 (m/z 120)

Ten TPs of p-aminobenzoic acid were detected, resulting from the degradation of any of the SAs present in the soil. SA_158, SA_174, SA_188, and SA_204 were formed by breaking the S-N bond in the SAs molecule and attaching a hydroxyl group to the sulfur atom. 4-aminobenzene-1-sulfonic acid (SA_174) was identified in six soil extracts and benzenesulfonic acid (SA_158) in 24 soil extracts. SA_174 and SA_158 are often detected in the abiotic (Kim et al. 2017; Liu et al. 2020) and biotic degradation processes of SAs (Kim et al. 2017; Wang and Wang 2018; Wang et al. 2018). The N-hydroxylation of 4-aminobenzenesulfonic acid formed SA_188. The SA_188 mass spectrum was acquired in ESI(-) mode, where the fragmentation ion with m/z 124 [M-H-SO_2_]^-^ was observed. SA_204 is formed by the oxidation of the SA_174 nitrogen atom to an NO_2_ group. The SA_204 fragmentation spectrum was consistent with those reported in the literature and had fragmentation ions with m/z 187 and 158 (Zhu et al. 2019; Deng et al. 2019). SA_219 was created due to the attachment of hydroxyl and carbonyl groups and the loss of aromaticity of the benzene ring of SA_174. SA_219 has been observed in the biodegradation process of SFP using *Trametes versicolor* fungi (Rodríguez-Rodríguez et al. 2012). Fragmentation ions with m/z 201 [M+H-H_2_O]^+^, m/z 176 [M+H-CH_2_NO]^+^, and 160 [M+H-CH_2_NO-OH]^+^ were present in the SA_219 mass spectrum. SA_173 and SA_187 are formed by breaking the N-C bond in the SA structure (Fig. 2 a). Both TPs were present in five soil samples (KBP, SWIII, KBS, SBP, and WLII), which may suggest that SA_173 may be a precursor to SA_187. SA_187 differs from SA_173 by having a methyl group attached to the sulfonamide group, and its mass spectrum was consistent with literature data (Cioroiu et al. 2013). The structure of SA_173 (4-aminobenzene-1-sulfonamide) was confirmed by the mass spectrum, where fragmentation ions with m/z 156 [M+H-NH_2_]^+^ and 109 [M+H-SO_2_]^+^ were observed. SA_120 is formed by the opening and cleavage of an aromatic benzene ring to form 2-but-2-ene-2-sulfinic acid. SA_120 was present in all soil samples where SAs residues were detected. The structure of SA_120 was confirmed by two characteristic p-MRM transitions from m/z 120 to m/z 91 and 65 (Fig. 2 b), which was consistent with the literature data (Al-Maqdi et al. 2018).

The degradation of p-aminobenzoic acid leads to simple benzene derivatives (BZ). BZ TPs are often formed in SAs photolysis reactions (Cioroiu et al. 2013; Zhu et al. 2019). BZ TPs were found in soil extracts, but it is impossible to establish their source. BZ_94 (aniline), BZ_111 (benzene-1,4-diol), and BZ_126 (4-aminobenzene-1,3-diol) can be formed through the degradation of SAs as well as other organic pollutants. BZ_111 was present in 4 soils, BZ_94 in 2 soils, and BZ_126 only in 1 soil, but KBP contained all three TPs. These BZs have been identified as TPs of SFT in the electro-Fenton process (Zhu et al. 2019), and the oxidation of SFD additionally forms BZ_94 (Cioroiu et al. 2013). 18 TPs were based on the backbone of aromatic amines present in the structures of the selected SAs. TPs were detected for seven of the eight selected SAs, except for SFH. SMX_208 is the only detected SMX TP, resulting from hydroxylation of the benzene ring, the opening of the isoxazole ring, and the loss of -SO_2_ group. SMX_208 was first identified by L. Zheng et al. in the electrochemical degradation of SMX (Zheng et al. 2019); however, this TP has been detected in our previous studies in surface waters (Kokoszka et al. 2021). Two SFD TPs were formed by N-carbonylation (SFD_279) and N-acetylation (SFD_293) reactions. SFD_279 and SFD_293 arise in biotic transformations of SFD and have been detected in pig feces, cabbage leaves, and surface waters (Lamshöft et al. 2007; Tian et al. 2019; Kokoszka et al. 2021). SFD_279, SFD_293, and SFD were present in 19 and 15 soil samples, respectively, suggesting that these TPs could be formed directly in the environment from the parent compound.

SFR differs from SFM by one methyl group attached to the pyrimidine ring, so their degradation may result in the formation of the same TPs. Both SAs were detected in 10 soil samples (SBS, OSPP, WLII, SWV, PPDG, SBP, SWIII, SPI, SPII, and KBP), so TPs were assigned to these compounds based on literature information (Babić et al. 2015; Li et al. 2017). SFM_124 (4,6-dimethylpyrimidin-2-amine) and SFM_140 (2-amino-4,6-dimethylpyrimidin-5-ol) are formed by the breakage of the N-C bond in the SFM structure. SFM_215 is created by opening a pyrimidine ring to form a diaminoimidomethane group. SFM_215 was identified based on retrospective analysis of the mass spectrum, in which fragmentation ions with m/z 198 and m/z 158 were present (Babić et al. 2015). All three TPs of SFM were detected in the mixture after the photodegradation of SFM (Babić et al. 2015; Li et al. 2017), but it cannot be ruled out that they may also be formed under environmental conditions.

**Fig. 3:**
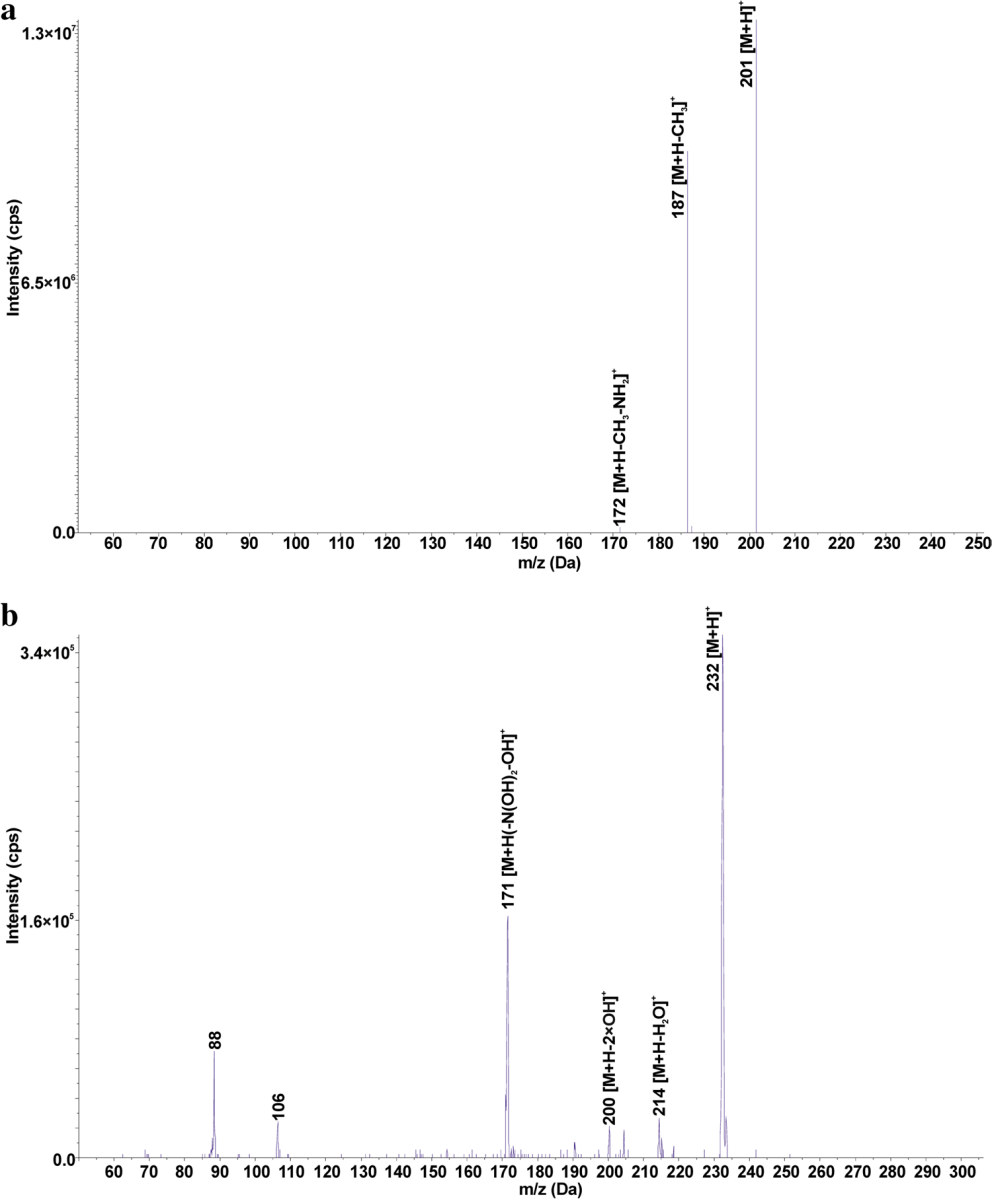
Mass spectra of the selected TPs obtained in EMS-IDA-EPI mode: a) SFR_201 (m/z 201) and b) SFP_232 (m/z 232)

Seven TPs of SFR were created due to the loss of the SO_2_ group (SFR_201), opening of the pyrimidine ring (SFR_216), hydroxylation (SFR_266, SFR_281, and SFR_297), and the breakage of the C-N bond (SFR_110 and SFR_169). The mass spectrum of SFR_201 is presented in Fig. 3 a. SFR_281 is formed by N-hydroxylation of the 4-aminobenzoic ring, while SFR_266 is produced by replacing an amino group with a hydroxyl group. SFR_297 is created by the dihydroxylation of SFR within the 4-aminobenzoate ring. All three hydroxylated TPs were detected in the Fenton water purification process (Deng et al. 2019; Chen et al. 2020). SFR_169 is formed by the oxidation of the amino and methyl groups in the structure of SFR_110. The structure of SFR_169 was confirmed based on fragmentation ions of m/z 141 [M+H-CO]^+^, 124 [M+H-NO_2_]^+^, and 81 [M+H-NO_2_-COOH]^+^ present in the mass spectrum. SFR_201, SFR_216, SFR_169, and SFR_110 were identified only in post-reaction mixtures after photocatalytic and electrochemical water treatment under laboratory conditions (Deng et al. 2019; Liu et al. 2020; Wang et al. 2020). The most frequently detected TPs of SFR in soils were SFR_281 (11), SFR_201 (10), SFR_266 (9), and SFR_297 (9). There is no information in the literature on the presence of hydroxylated SFR TPs in the soil-water environment. Hydroxylation is a reaction characteristic of the first phase of drug metabolism (Huynh and Reinhold 2019), and we suppose that these compounds can also be formed under environmental conditions.

SFX_113, SFP_95, and SFT_101 are formed by cleavage of the S-N bond in the molecule of the corresponding SA. As a result of breaking the S-C bond in the SFT molecule and then attaching the hydroxyl group to the sulfur atom, SFT_181 is formed. SFP_232 is created by the removal of SO_2_ and the triple N-hydroxylation of SFP (Fig. 3b). SFP_232 was present in all soils where SFP was detected, suggesting that it may arise under environmental conditions from the parent SA. SFT_101 and SFT_181 were detected only in single soils (PPDG and KBP, respectively), so it was assumed that they were not caused by the degradation of SFT in soil. In the literature, the mentioned TPs of SFX, SFP, and SFT were detected in post-reaction mixtures after advanced oxidation processes (Yao et al. 2017; Zhu et al. 2019).

## Conclusions

A universal, effective, reproducible method of extracting eight SAs from soil samples with different physicochemical properties was developed as part of this research. A two-step SLE-SPE extraction procedure was used to isolate and concentrate the analytes. The SAs recovery from the soil matrix was strongly dependent on the OC level, and a significant decrease in recoveries was observed for OC>2%. The recovery of selected SAs depended to a varying extent on the content of sodium and potassium in the soil, but no effect of pH was observed. It was noted that with an increase in the OC, K, or Na content, SAs bound more strongly to soil particles, making them difficult to extract. To confirm the universality of the method, the SAs recovery was determined at four levels of OC content. Based on the validation parameters, it was found that the developed SLE-SPE-LC-MS/MS method was sensitive, accurate, and precise, so it was used to determine SAs in 27 types of soil samples.

The soil samples were collected from the areas of six cities in the highly urbanized agglomeration in Silesian Voivodeship. The sampling sites were selected using the following criteria: increased soil contact with the livestock (agricultural fields) or domestic animals (parks, paddocks, or tourist resorts). Each soil sample was characterized in terms of the content of OC, Al, Ca, Mg, Na, K, and pH. Each of the eight SAs was detected in the soil samples. The most frequently detected SAs were SMX (23 soils) and SFD (19 soils). The highest concentrations of SA were found in soils from dog paddocks (1.7–10.5 ng g^-1^) and agricultural fields (1.9–3.7 ng g^-1^). It is worth noting that the content of SAs in the soils from the dog runs was higher than in the agricultural soils. This observation suggests that the increased number and activity of dogs in their designated areas might be a contributing factor to the higher presence of SAs in the soil. The screening revealed 29 SAs TPs resulting from the transformation of 4-aminobenzoic acid or an aromatic amine that was part of the structure of an SA. TPs were detected in 24 out of 27 soil samples, and the highest amount was detected in soil from agricultural fields (KBP, 20 TPs) and dog paddocks (SPII, 17 TPs; WL, 16 TPs; SWIII, 15 TPs). This suggests that the formation of TPs in soil depends on the concentration of SAs in the soil and on the frequency of their introduction into the environment. TPs were formed by hydroxylation, ring opening, and the breaking of S-N, N-C, and S-C bonds. To the best of our knowledge, this is one of the few studies dealing with TPs in the community (Hoff et al. 2016), so the comparison of our data with the literature is difficult.

According to our knowledge, this is the first paper to consider the effect of soil OC content on SAs recovery. The impact of OC on recovery is crucial in comparing SAs residues in different soil types. The obtained results suggest that SAs are constantly being transferred to the environment, including in highly urbanized areas. It has been found that excrement from veterinary-treated domestic animals may also be a source of soil contamination with SAs. The findings reported here fill a research gap on the spread of pharmaceuticals in urban areas which can also be a potential reservoir for the emergence of drug-resistant bacterial strains.

### Supplementary Information

ESM 1 (DOCX 207 kb)

## Data Availability

All data generated and analyzed during our study are included in this article.
