# Supplementary material for: Application of UHPLC-MS/MS method to monitor the occurrence of sulfonamides and their transformation products in soil in Silesia, Poland
Source: Environ Sci Pollut Res Int. 2023 Oct 16;30(52):112922–42. doi: 10.1007/s11356-023-30146-y (PMC10643288; doi:10.1007/s11356-023-30146-y)
Supplement: Supplementary file 1 — (DOCX 207 kb) [file 11356_2023_30146_MOESM1_ESM.docx]

**Application of UHPLC-MS/MS method to monitor the occurrence of sulfonamides and their transformation products in soil in Silesia, Poland**

Klaudia Stando^1^, Joanna Wilk^1^, Agata Jakóbik-Kolon^1^, Ewa Felis^2,3^, Sylwia Bajkacz^1,2^

*^1^ Silesian University of Technology, Faculty of Chemistry, Department of Inorganic Chemistry, Analytical Chemistry and Electrochemistry, B. Krzywoustego 6 Str., 44-100 Gliwice, Poland*

*^2^ Silesian University of Technology, Biotechnology Centre, B. Krzywoustego 8 Str, 44-100 Gliwice, Poland*

*^3^ Silesian University of Technology, Faculty of Power and Environmental Engineering, Environmental Biotechnology Department, Akademicka 2 Str., 44-100 Gliwice, Poland*

**SUPPLEMENTARY MATERIAL**

**Table S1.** Comparison of changed parameters of tested SLE procedures (1-7)

**Table S2.** Comparison of changed parameters of tested SPE procedures (A-H)

**Table S3.** Optimized MRM parameters for target SAs

**Table S4.** Recoveries obtained for examined SLE-SPE procedures (blank sample used as a model sample)

**Table S5.** Physicochemical properties of soil samples collected in Silesia

**Table S6.** Distribution of SAs and their TPs in soils

**Figure S1.** Comparison of recoveries obtained for SLE7+SPE8 procedure conducted for arenaceous quartz (OC<LOQ), and blank sample used as a model sample (OC=3.02%)

Table S1. Comparison of changed parameters of tested SLE procedures (1-7)

| No. | Solvent | Sonication (min) | Shaking (min) | Centrifugation |
| --- | --- | --- | --- | --- |
| SLE1 | 10 mL McIlvaine buffer pH 4 + MeOH (1:1; V/V) | 10 | 30 | 10 min  8000 rpm |
| SLE2 | 1) 10 mL McIlvaine buffer pH 4 + MeOH (1:1; V/V)  2) 10 mL McIlvaine buffer pH 4 + ACN (1:1; V/V) | 10 | 30 |  |
| SLE3 |  | - | 60 |  |
| SLE4 | 1) 10 mL McIlvaine buffer pH 4 + MeOH (1:1; V/V)  2) 10 mL McIlvaine buffer pH 4 + ACN (1:1; V/V)  + 0.1 g NaF | 10 | 30 |  |
| SLE5 | 1) 10 mL 0.1 M EDTA + McIlvaine buffer pH 4 (1:1; V/V)  2) 10 mL MeOH + ACN + Ace (2:2:1; V/V/V) | 10 | 30 |  |
| SLE6 | 2 × 10 mL MeOH + ACN + 0.1 M EDTA +  McIlvaine buffer pH 4 (30:20:25:25; V/V/V/V) | 10 | 30 |  |
| SLE7 |  | - | 60 |  |

*ACN – acetonitrile, Ace – acetone, MeOH – methanol, EDTA - ethylenediaminetetraacetic acid*

Table S2. Comparison of changed parameters of tested SPE procedures (A-H)

| Tag | SPE cartridge | Conditioning solvent | pH | Elution solvent | Remainings reconstitution |
| --- | --- | --- | --- | --- | --- |
| SPE1 | Oasis HLB  (500 mg, 6 mL) | 6 mL MeOH, 6 mL 0.1 M HCl,  6 mL H_2_O | 4 | 12 mL MeOH | 1 mL MeOH |
| SPE2 | Oasis HLB  (500 mg, 6 mL) | 6 mL MeOH, 6 mL 0.1 M HCl,  6 mL H_2_O | 3 | 12 mL MeOH | 1 mL MeOH |
| SPE3 | Oasis MAX  (150 mg, 6 mL)  Oasis HLB  (500 mg, 6 mL) | 6 mL MeOH, 6 mL 0.1 M HCl,  6 mL H_2_O | 4 | 12 mL MeOH | 1 mL MeOH |
| SPE4 | Oasis HLB  (500 mg, 6 mL) | 6 mL MeOH, 6 mL H_2_O,  6 mL McIlvaine buffer pH=4 | 4 | 12 mL MeOH | 1 mL MeOH |
| SPE5 | Oasis HLB  (500 mg, 6 mL) | 6 mL MeOH, 6 mL H_2_O,  6 mL McIlvaine buffer pH=4 | 4 | 12 mL MeOH | 1 mL MeOH  Washing: MeOH + H_2_O (2:3; V/V) |
| SPE6 | Oasis HLB  (500 mg, 6 mL) | 6 mL MeOH, 6 mL H_2_O,  6 mL McIlvaine buffer pH=4 | 4 | 12 mL MeOH | Eluate dried under N_2_ to 1 mL |
| SPE7 | Oasis HLB  (500 mg, 6 mL) | 6 mL MeOH, 6 mL H_2_O,  6 mL McIlvaine buffer pH=4 | 4 | 12 mL 0.1% FA in MeOH | Eluate dried under N_2_ to 1 mL |
| SPE8 |  |  |  | 12 mL 0.1% AcA in MeOH |  |

*FA – formic acid, AcA – acetic acid*

Table S3. Optimized MRM parameters for target SAs

| Abbrev. | Q1 (m/z) | Q3 (m/z) | DP (V) | CE (V) | CXP (V) | EP (V) |
| --- | --- | --- | --- | --- | --- | --- |
| SMX | 253.7 | 108.1  156.1 | 71 | 35  23 | 8  10 | 7 |
| SFH | 270.8 | 156.1  92.1 | 71 | 81  37 | 12  6 | 7 |
| SFT | 255.8 | 156.2  92.2 | 71 | 21  39 | 12  6 | 7 |
| SFP | 249.9 | 92.2  156.1 | 81 | 43  5 | 6  14 | 7 |
| SFD | 250.9 | 108.1  157.0 | 71 | 33  23 | 8  12 | 7 |
| SFM | 278.8 | 186.2  124.2 | 71 | 83  35 | 4  8 | 7 |
| SFR | 264.9 | 156.1  92.2 | 51 | 25  43 | 12  6 | 7 |
| SFX | 267.8 | 156.2 113.1 | 71 | 21  23 | 10  8 | 7 |

Table S4. Recoveries obtained for examined SLE-SPE procedures (blank sample used as a model sample)

| **SA** | **Procedure** | | | | | | | | | | |
| --- | --- | --- | --- | --- | --- | --- | --- | --- | --- | --- | --- |
|  | SLE1+ SPE1 | SLE2+ SPE1 | SLE2+ SPE2 | SLE4+ SPE1 | SLE5+ SPE1 | SLE6+ SPE4 | SLE2+ SPE3 | SLE3+ SPE5 | SLE7+ SPE6 | SLE7+ SPE7 | SLE7+ SPE8 |
|  | **R (%)** | | | | | | | | | | |
| SMX | 29.5 | 28.3 | 26.6 | 35.6 | 31.9 | 42.1 | 0.2 | 8.9 | 63.3 | 53.6 | 78.4 |
| SFH | 20.0 | 22.8 | 30.4 | 16.1 | 25.5 | 39.1 | 0.0 | 10.1 | 54.8 | 29.1 | 45.7 |
| SFT | 17.5 | 23.0 | 19.1 | 14.0 | 14.5 | 19.5 | 2.1 | 9.7 | 40.9 | 28.8 | 50.9 |
| SFP | 19.7 | 25.0 | 11.9 | 16.5 | 8.2 | 13.5 | 6.9 | 11.7 | 44.9 | 21.0 | 47.5 |
| SFD | 25.7 | 28.5 | 30.1 | 20.5 | 25.0 | 35.0 | 16.0 | 8.9 | 56.1 | 41.6 | 60.6 |
| SFM | 22.5 | 26.7 | 26.6 | 16.7 | 18.9 | 36.9 | 2.0 | 13.5 | 50.0 | 32.6 | 49.9 |
| SFR | 22.9 | 28.1 | 29.4 | 19.9 | 22.4 | 33.1 | 5.7 | 14.2 | 66.5 | 42.5 | 61.4 |
| SFX | 18.2 | 21.1 | 16.0 | 23.7 | 16.6 | 19.5 | 0.1 | 17.2 | 56.1 | 45.3 | 58.5 |
|  | **SD (%)** | | | | | | | | | | |
| SMX | 17.8 | 3.8 | 1.3 | 4.8 | 1.0 | 6.4 | 0.1 | 0.6 | 5.4 | 4.9 | 5.7 |
| SFH | 11.5 | 1.0 | 2.8 | 4.0 | 2.0 | 2.9 | 0.0 | 1.0 | 7.1 | 1.4 | 3.1 |
| SFT | 11.8 | 2.6 | 1.6 | 2.8 | 1.1 | 3.7 | 1.0 | 1.3 | 7.3 | 1.1 | 2.9 |
| SFP | 14.5 | 3.2 | 0.8 | 5.5 | 0.9 | 2.0 | 3.3 | 1.3 | 7.9 | 1.6 | 3.4 |
| SFD | 18.7 | 0.9 | 3.0 | 4.9 | 0.5 | 0.8 | 7.1 | 2.8 | 6.3 | 1.3 | 3.8 |
| SFM | 18.0 | 2.0 | 2.0 | 3.3 | 2.0 | 1.4 | 1.0 | 1.2 | 8.7 | 1.1 | 2.6 |
| SFR | 17.2 | 1.9 | 2.2 | 4.5 | 1.9 | 1.1 | 2.5 | 1.2 | 9.4 | 3.1 | 4.1 |
| SFX | 12.8 | 1.9 | 0.5 | 4.0 | 0.1 | 1.7 | 0.1 | 0.9 | 6.8 | 1.8 | 3.1 |
|  | **CV (%)** | | | | | | | | | | |
| SMX | 60.2 | 13.6 | 5.0 | 13.5 | 3.3 | 15.2 | 68.6 | 28.9 | 8.6 | 9.2 | 7.3 |
| SFH | 57.1 | 22.8 | 9.1 | 25.0 | 8.0 | 7.5 | 56.8 | 32.7 | 13.0 | 4.7 | 6.8 |
| SFT | 67.6 | 11.3 | 8.3 | 20.3 | 7.2 | 19.0 | 44.8 | 34.3 | 17.9 | 3.9 | 5.7 |
| SFP | 73.5 | 12.7 | 7.1 | 33.5 | 10.6 | 15.1 | 47.4 | 28.6 | 17.6 | 7.5 | 7.2 |
| SFD | 72.6 | 3.2 | 10.0 | 24.2 | 2.0 | 44.3 | 2.2 | 87.0 | 11.3 | 3.2 | 6.2 |
| SFM | 79.9 | 7.4 | 7.4 | 19.8 | 10.5 | 48.6 | 3.9 | 27.1 | 17.5 | 3.2 | 5.2 |
| SFR | 75.3 | 6.8 | 7.6 | 22.8 | 8.5 | 43.9 | 3.3 | 24.6 | 14.2 | 7.4 | 6.7 |
| SFX | 70.7 | 9.1 | 3.2 | 16.8 | 0.8 | 83.5 | 8.7 | 21.5 | 12.1 | 3.9 | 5.3 |

Table S5. Physicochemical properties of soil samples collected in Silesia

| Abbrev. | Locality-sampling place | OC±SD (%) | pH | C_Al_±SD (mg g^-1^) | C_Ca_±SD (mg g^-1^) | C_Mg_±SD (mg g^-1^) | C_Na_±SD (mg g^-1^) | C_K_±SD (mg g^-1^) |
| --- | --- | --- | --- | --- | --- | --- | --- | --- |
| CPDG | Dąbrowa Górnicza – Pogoria III camping centre | <LOQ | 8.06 | 4.37±0.21 | 1.50±0.12 | 0.66±0.02 | 0.05±0.01 | 0.63±0.02 |
| PPDG | Dąbrowa Górnicza – Pogoria III beach | <LOQ | 7.50 | 2.38±0.07 | 0.67±0.11 | 0.31±0.03 | <LOQ | 0.31±0.01 |
| ŚPDG | Dąbrowa Górnicza – Pogoria III tourist place | <LOQ | 7.85 | 3.98±0.44 | 1.39±0.31 | 0.61±0.07 | <LOQ | 0.52±0.05 |
| PZDG | Dąbrowa Górnicza – “Zielona” park | 2.54±0.19 | 7.38 | 11.32±0.48 | 7.72±0.38 | 2.43±0.30 | 0.18±0.03 | 1.22±0.09 |
| SBP | Katowice – Borki lake beach | <LOQ | 8.49 | 3.32±0.26 | 0.38±0.01 | 0.32±0.02 | <LOQ | 0.34±0.03 |
| SBŚ | Katowice – Borki lake tourist place | 2.14±0.60 | 8.49 | 6.40±1.15 | 2.26±0.31 | 0.84±0.18 | 0.10±0.03 | 0.64±0.13 |
| WLI | Katowice Ligota – Dog paddock I | 4.12±0.70 | 7.44 | 7.29±0.32 | 10.01±0.90 | 1.21±0.07 | 0.10±0.01 | 0.90±0.06 |
| WLII | Katowice Ligota – Dog paddock II | 1.76±0.21 | 7.17 | 6.99±0.069 | 2.85±0.38 | 0.75±0.10 | 0.06±0.01 | 0.72±0.06 |
| KBS | Katowice Burowiec – Horse paddock | 4.23±0.18 | 6.05 | 13.28±0.68 | 3.56±0.27 | 1.97±0.11 | 0.11±0.01 | 1.36±0.14 |
| KBP | Katowice Burowiec – Agricultural field | 2.61±0.56 | 7.63 | 12.86±0.48 | 2.99±0.17 | 1.34±0.05 | 0.11±0.02 | 1.24±0.04 |
| SKM | Mikołów– Horse paddock | 1.71±0.12 | 8.57 | 11.13±0.77 | 2.85±0.44 | 1.61±0.22 | 0.17±0.02 | 1.35±0.10 |
| PM | Mikołów –Agricultural field | 2.30±0.07 | 8.80 | 10.23±0.43 | 4.49±0.50 | 1.43±0.18 | 0.11±0.01 | 0.98±0.16 |
| PP | Przyłęk – Agricultural field | 2.44±0.11 | 6.80 | 5.89±0.36 | 1.08±0.08 | 0.58±0.03 | <LOQ | 0.86±0.06 |
| SWI | Sosnowiec – Dog paddock I | 3.21±0.30 | 6.56 | 13.21±0.33 | 6.72±0.37 | 1.75±0.05 | 0.25±0.01 | 1.68±0.05 |
| SWII | Sosnowiec – Dog paddock II | 3.98±0.59 | 6.71 | 7.86±0.29 | 4.57±0.48 | 1.66±0.02 | 0.10±0.01 | 0.84±0.02 |
| SWIII | Sosnowiec – Dog paddock III | 1.66±0.50 | 7.19 | 7.15±1.15 | 6.81±0.69 | 1.40±0.19 | 0.08±0.01 | 0.91±0.16 |
| SWIV | Sosnowiec – Dog paddock IV | 2.72±0.28 | 7.25 | 11.61±0.35 | 6.18±0.39 | 1.56±0.09 | 0.11±0.00 | 1.25±0.04 |
| SWV | Sosnowiec – Dog paddock V | 2.61±0.21 | 8.73 | 11.39±0.17 | 17.72±0.08 | 3.30±0.15 | 0.19±0.03 | 1.51±0.01 |
| SPI | Sosnowiec – Unfenced dog paddock I | 4.91±0.75 | 6.23 | 7.97±0.96 | 6.09±0.94 | 1.81±0.28 | 0.13±0.02 | 1.25±0.14 |
| SPII | Sosnowiec – Unfenced dog paddock II | 2.39±0.30 | 6.81 | 8.91±0.63 | 5.25±0.84 | 1.28±0.10 | 0.08±0.01 | 1.25±0.12 |
| SWET | Sosnowiec – Veterinary dog paddock III | 1.50±0.15 | 7.58 | 6.52±0.88 | 4.93±0.38 | 1.45±0.01 | 0.09±0.02 | 0.81±0.18 |
| OSPP | Tychy – Paprocany lake beach | <LOQ | 6.55 | 2.10±0.27 | 0.29±0.05 | 0.26±0.05 | <LOQ | 0.33±0.05 |
| JKI | Tychy – Northen Park lake shore I | 5.30±0.03 | 5.45 | 15.86±0.77 | 2.71±0.35 | 1.51±0.16 | 0.36±0.08 | 1.60±0.06 |
| JKII | Tychy – Northen Park lake shore II | 4.36±0.12 | 5.13 | 15.04±0.36 | 1.90±0.19 | 1.38±0.05 | 0.14±0.01 | 1.56±0.02 |
| PCENT | Tychy – Paprocany tourist center | 2.23±0.50 | 7.30 | 9.51±0.59 | 9.41±0.46 | 5.40±0.35 | 0.07±0.02 | 1.41±0.10 |
| DPP | Tychy – Paprocany “wild beach” | <LOQ | 6.44 | 2.61±0.55 | 0.26±0.09 | 0.38±0.13 | <LOQ | 0.33±0.07 |
| PN | Tychy – “Bear cub” park | 4.70±0.56 | 5.90 | 12.64±0.23 | 2.29±0.80 | 1.47±0.12 | 0.11±0.00 | 1.48±0.11 |

*LOQ values: OC=1.07%. C_Al_=C_Ca_=C_Mg_=C_K_=0.01 mg* g^-1^

Table S6. Distribution of SAs and their TPs in soils

| **Abbrevations** | **PM** | **SBS** | **JK1** | **JK2** | **DPP** | **OsPP** | **WLII** | **WLI** | **SWV** | **PN** | **PPDG** | **SBP** | **PP** | **KBS** | **SWIII** | **PCENT** | **SP I** | **SW II** | **SPDG** | **SKM** | **SWET** | **SWI** | **SW IV** | **CPDG** | **SP II** | **PZDG** | **KBP** | **Frequency** |
| --- | --- | --- | --- | --- | --- | --- | --- | --- | --- | --- | --- | --- | --- | --- | --- | --- | --- | --- | --- | --- | --- | --- | --- | --- | --- | --- | --- | --- |
| SMX | + | + | - | + | + | + | + | + | + | + | + | + | + | + | + | + | + | + | - | + | - | + | + | - | + | + | + | 23 |
| SFH | - | + | - | - | + | + | + | + | + | - | + | + | - | - | + | + | + | - | - | - | - | - | - | - | + | - | + | 13 |
| SFT | - | - | - | - | - | - | + | + | - | - | + | + | - | + | + | - | - | - | - | - | - | - | - | - | - | - | + | 7 |
| SFP | - | + | - | - | - | + | + | + | + | - | - | - | - | - | + | + | + | - | - | - | - | - | - | - | + | - | + | 10 |
| SFD | + | + | - | - | + | + | + | + | + | - | + | + | - | - | + | + | + | + | - | - | + | + | + | - | + | + | + | 19 |
| SFM | - | + | - | - | - | + | + | + | + | - | + | + | - | - | + | + | + | - | - | - | - | - | - | - | + | - | + | 12 |
| SFR | - | + | - | - | + | + | + | - | + | - | + | + | - | - | + | - | + | - | - | - | - | - | - | - | + | - | + | 11 |
| SFX | - | + | - | - | - | + | + | - | - | - | - | + | - | - | + | - | + | - | - | - | - | - | - | - | + | - | + | 8 |
| SA_120 | + | + | - | + | + | + | + | + | + | + | + | + | + | + | + | + | + | + | - | + | + | + | + | - | + | + | + | 24 |
| SA_158 | + | + | - | + | + | + | + | + | + | + | + | + | + | + | + | + | + | + | - | + | + | + | + | - | + | + | + | 24 |
| SA_173 | - | - | - | - | - | - | + | - | - | - | - | + | - | + | + | - | - | - | - | - | - | - | - | - | - | - | + | 5 |
| SA_174 | - | - | - | - | + | + | - | - | + | - | - | - | - | - | - | - | + | - | - | - | - | - | - | - | + | - | + | 6 |
| SA_187 | + | + | - | + | - | - | + | + | + | + | + | + | - | + | + | + | + | + | - | + | + | + | + | - | + | + | + | 21 |
| SA_188 | - | + | - | - | - | - | - | - | - | - | - | - | - | - | + | - | - | - | - | - | - | - | - | - | + | - | + | 4 |
| SA_204 | - | - | - | - | - | - | - | - | - | - | - | - | - | - | + | - | - | - | - | - | - | - | - | - | - | - | - | 1 |
| SA_219 | - | + | - | - | - | - | - | - | - | - | - | - | - | - | + | - | - | - | - | - | - | - | - | - | - | - | - | 2 |
| BZ_94 | - | - | - | - | - | - | + | - | - | - | - | - | - | - | - | - | - | - | - | - | - | - | - | - | - | - | + | 2 |
| BZ_111 | - | - | - | - | - | - | + | - | - | - | - | + | - | - | + | - | - | - | - | - | - | - | - | - | - | - | + | 4 |
| BZ_126 | - | - | - | - | - | - | - | - | - | - | - | - | - | - | - | - | - | - | - | - | - | - | - | - | - | - | + | 1 |
| SMX_208 | - | - | - | - | - | - | - | - | + | - | - | + | - | - | - | - | - | - | - | - | - | - | - | - | + | - | - | 3 |
| SFD_293 | + | + | - | - | + | + | + | + | + | - | + | + | - | - | + | + | + | + | - | - | + | + | + | - | + | + | + | 19 |
| SFD_279 | + | - | - | + | - | - | + | - | - | + | - | + | - | - | + | + | + | + | - | - | + | + | + | - | + | + | + | 15 |
| SFM_140 | - | - | - | - | - | - | - | - | + | - | - | - | - | - | - | + | - | - | - | - | - | - | - | - | - | - | + | 3 |
| SFM_215 | - | - | - | - | - | - | + | - | + | - | - | - | - | - | - | + | - | - | - | - | - | - | - | - | + | - | + | 5 |
| SFM_124 | - | - | - | - | - | - | - | - | + | - | - | + | - | - | - | - | - | - | - | - | - | - | - | - | - | - | - | 2 |
| SFR_110 | - | + | - | - | - | - | - | - | - | - | - | - | - | - | - | - | - | - | - | - | - | - | - | - | - | - | - | 1 |
| SFR_169 | - | - | - | - | - | + | - | - | - | - | - | - | - | - | - | - | - | - | - | - | - | - | - | - | + | - | - | 2 |
| SFR_201 | - | + | - | - | + | + | + | - | - | - | + | + | - | - | + | - | + | - | - | - | - | - | - | - | + | - | + | 10 |
| SFR_216 | - | - | - | - | - | - | - | - | - | - | - | - | - | - | - | - | - | - | - | - | - | - | - | - | + | - | - | 1 |
| SFR_266 | - | + | - | - | - | - | + | - | + | - | + | + | - | - | + | - | + | - | - | - | - | - | - | - | + | - | + | 9 |
| SFR_281 | - | + | - | - | + | + | + | - | + | - | + | + | - | - | + | - | + | - | - | - | - | - | - | - | + | - | + | 11 |
| SFR_297 | - | + | - | - | + | + | + | - | - | - | - | + | - | - | + | - | - | - | - | - | - | - | - | - | + | + | + | 9 |
| SFX_113 | - | + | - | - | - | + | + | - | - | - | - | - | - | - | - | - | - | - | - | - | - | - | - | - | + | - | - | 4 |
| SFP_95 | - | + | - | - | - | + | + | + | - | - | - | - | - | - | - | - | - | - | - | - | - | - | - | - | - | - | + | 5 |
| SFP_232 | - | + | - | - | - | + | + | + | + | - | - | - | - | - | + | + | + | - | - | - | - | - | - | - | + | - | + | 10 |
| SFT_181 | - | - | - | - | - | - | - | - | - | - | + | - | - | - | - | - | - | - | - | - | - | - | - | - | - | - | - | 1 |
| SFT_101 | - | - | - | - | - | - | - | - | - | - | - | - | - | - | - | - | - | - | - | - | - | - | - | - | - | - | + | 1 |
| Σ_SAs_ | 2 | 7 | 0 | 1 | 4 | 7 | 8 | 6 | 6 | 1 | 6 | 7 | 1 | 2 | 8 | 5 | 7 | 2 | 0 | 1 | 1 | 2 | 2 | 0 | 7 | 2 | 8 |  |
| Σ_TPs_ | 5 | 14 | 0 | 4 | 7 | 11 | 16 | 6 | 12 | 4 | 8 | 13 | 2 | 4 | 15 | 8 | 10 | 5 | 0 | 3 | 5 | 5 | 5 | 0 | 17 | 6 | 20 |  |


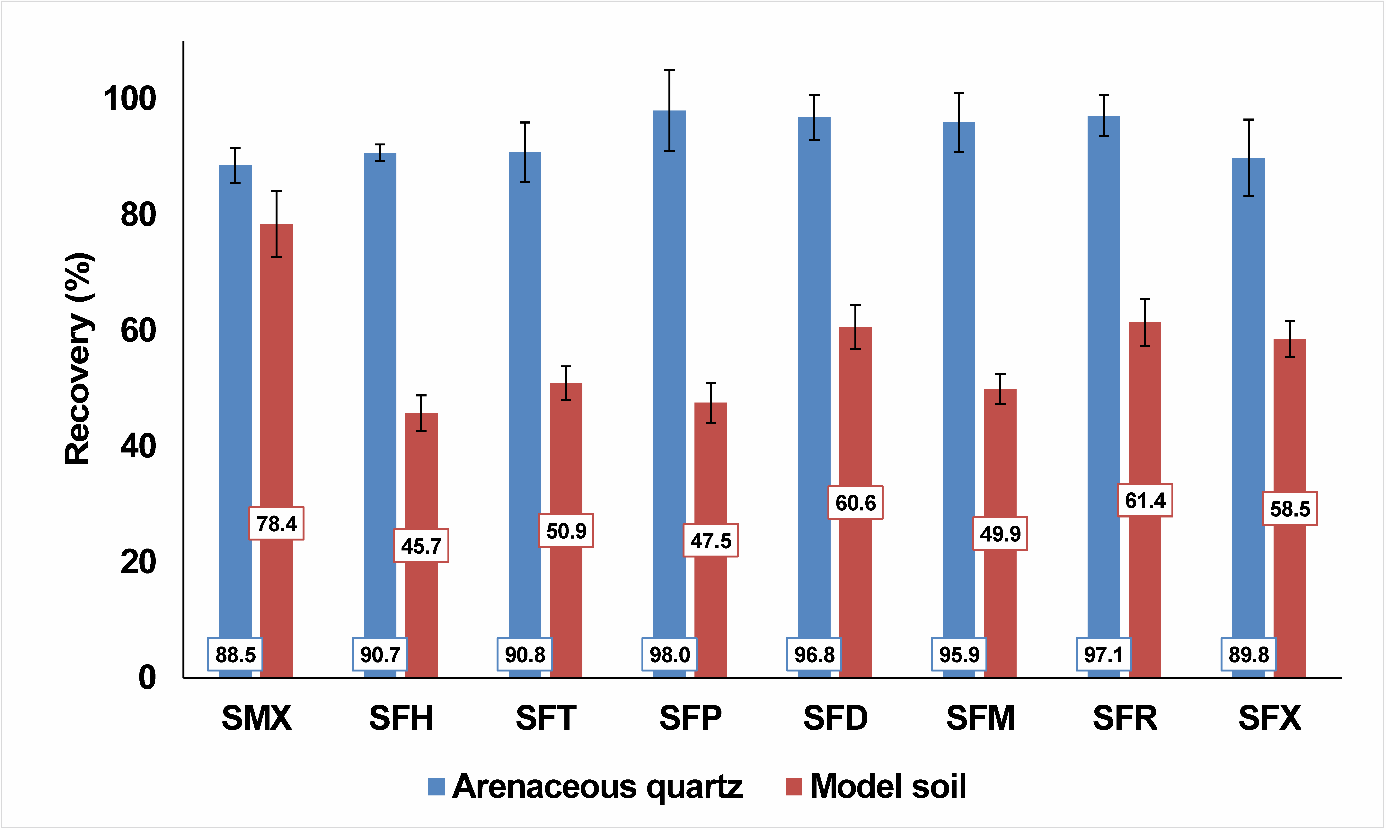


Fig. S1. Comparison of recoveries obtained for SLE7+SPE8 procedure conducted for arenaceous quartz (OC<LOQ), and blank sample used as a model sample (OC=3.02%)
